# Supplementary material for: Analysis of Biologics Molecular Descriptors towards Predictive Modelling for Protein Drug Development Using Time-Gated Raman Spectroscopy
Source: Pharmaceutics. 2022 Aug 5;14(8):1639. doi: 10.3390/pharmaceutics14081639 (PMC9413954; doi:10.3390/pharmaceutics14081639)
Supplement: Supplementary file 1 [file pharmaceutics-14-01639-s001.zip › pharmaceutics-1624751-supplementary.pdf]

Analysis of biologics molecular descriptors towards predictive modelling for protein drug development using time-gated Raman spectroscopy

Jaakko Itkonen [1,†], Leo Ghemtio [1,†], Daniela Pellegrino [1], Pia J. Jokela (née Heinonen) [1,2], Henri Xhaard [3] and Marco G. Casteleijn [4,*]

Supplementary data

Table S1. Specific protein band assignments for different protein classes with a 532 nm laser; adapted from Rygula *et al.* (2013) [1] and De Gelder *et al.* [2].

	Wavenumber [cm^{-1}]		
**Bond type**	**Alpha Helix**	**Beta sheet**	**Alpha / Beta**
Amide I	1657	1669	1664
**Tyr,Trp,Phe**	**1610**	**1619**	**1621**
Indole ring (Trp)	-	1553	-
**C-H (def)**	**1454**	**1453**	**1482**
C-H (def)	-	-	1453
**Trp, Cα-H (def)**	**1341**	**1342**	**-**
Trp, Cα-H (def)	1321	-	-
**Amide II**	**1273**	**1236**	**1283**
Amide III	1246	-	1236
**N-acetyl mannose**		**1139**	
C-N	1130	1123	1144
**N-acetyl fucose**		**1129**	
N-acetyl glucosamine		1126	
**Phe**	**1036**	**1036**	**1035**
Phe	1009	1007	1004
**N-Cα-C**	**945**	**945**	**935**
Trp	880	883	-
**Tyr**	**856**	**856**	**861**
Tyr	832	830	847
**Trp**	**755**	**764**	**-**
Tyr	651	647	-
**S-S**	**512**	**545**	**-**

Table S2. Parameters of Time-gated spectroscopy thermo-ramping.

Ramping starting temperature [° C]	Ramping rate [° C/min][a]	Ramping time [min]	Holding time [min] (time-gated scan)
20	2.5	4	6.05
30	2.0	3.5	6.05
37	1.5	2	6.05
40	1.7	3	6.05
45	1.7	3	6.05
50	1.7	3	6.05
52	0.7	3	6.05
57	1.7	3	6.05
60	1.0	3	6.05
65	1.7	3	6.05
70	1.7	3	6.05
75	1.7	3	6.05
80	1.7	3	6.05
85	-	-	6.05

(a) The average ramping speed was 1.6 °C/min.

Table S3. Identification of significant peak changes for BSA.

Significant peaks identified with PC1 [cm^{-1}]	Bond type	Significant peaks identified with PC2 [cm^{-1}]	Bond type	Peaks identified with PC2 [cm^{-1}]	Bond type
**880-900**	Trp	470	S-S stretch	835	Tyr
**910**	Ser[a]	495	S-S	1005	Phe
**940**	N-Cα-C	510	S-S	1130	C-N
**950**	N-Cα-C	550	S-S	1150	Tyr/Phe/ Trp[a]
**970**	Ser/His[a]	590	S-S	1180	Tyr/Phe/ Trp[a]
**1180**	Val/Arg/other amino acids[a]	600	Trp[a]	1200-1210	Phe, Tyr[a]
**1280**	Amide II	640	Tyr	1240	Amide III (β)
**1310**	Phe, Tyr, Trp[a]	680	Pro/His/Trp[a]	1250	Amide III (random coil)
		700	Trp[a]	1480	C-H (α/β)
		750	Trp/Val/Phe[a]	1500	His/Ala/ Gly[a, b]
		780	Glu/Val/Trp/ His[a]	1550	Indole ring
		800	His/Glu[a]	1550-1720	Amide I

(a) According to De Gelder et al. (2007) [2]. (b) Likely changes in the backbone (N-Cα-C).

Table S4. Identification of significant peak changes for pepsin.

**Significant peaks identified with PC1 [cm^{-1}]**	**Bond type**	**Significant peaks identified with PC2 [cm^{-1}]**	**Bond type**	**Peaks identified with PC2 [cm^{-1}]**	**Bond type**
**1390**	Trp	490	S-S	1080	His, Pro, Ser, Glu, Trp[a]
**1520**	Ser[a]	502	S-S	1100	C-N
**1540-1720**	Amide I	550	S-S	1190	Tyr/Phe/ Trp[a]
**1760**	Carbonyl stretching	660-670	Glu, Val[a]	1230	Amide II (β)
		840	Tyr	1250	Amide III (random coil)
		860	Tyr	1270-1330	Trp?
		1000	Phe	1360	C-N
		1030	Phe	1400	Ala, Val, Gly, Glu, Trp[a,b]
		1060	Tyr/Phe/ Trp[a]	1420	Val, Ser, Glu, Trp, Pro[a]

(a) According to De Gelder et al. (2007) [2]. (b) Likely changes in the backbone (N-Cα-C).

Table S5. Identification of significant peak changes for IgG .

**Significant peaks identified with PC1 (glyco) [cm^{-1}]**	**Bond type**	**Significant peaks identified with PC1 (non-glyco) [cm^{-1}]**	**Bond type**	**Peaks identified with PC2 [cm^{-1}][c]**	**Bond type**
**520-575**	S-S stretch	620	S-S	910 (g)	Ser[a]
**585-835**	S-S/Trpr/Tyr/Pro/His/Trp[a]	650	S-S	960 (g)	Ser/His[a]
**925**	Ala, Ser, Val, Arg, His[a,b]	760	Trp/Val/Phe[a]	1120 (g)	N-acetyl glucosamine/C-N
**940-1030**	Phe	820	Tyr, Val[a]	1235 (ng)	Amide II (β)
**1160**	Glu, Phe, Trp[a]	900-940	N-Cα-C	1400 (g)	Ala, Val, Gly, Glu, Trp[a,b]
**1220**	Amide II (β)	1040	Phe		
**1310**	Phe,Tyr, Trp[a]	1055	Tyr/Phe/ Trp[a]		
**1320**	Val/Glu/Trp[a]	1115	C-N		
**1350**	Trp[a]	1250	Amide II (random coil)		
**1700-1850**	Amide I / C=O stretch	1530	Indole ring		
		1610	Tyr,Trp,Phe		
		1695	Amide I (antiparallel β-sheet)		

(a) According to De Gelder *et al.* (2007) [2]. (b) Likely changes in the backbone (N-Cα-C). (c) g: glycosylated IgG; ng: non-glycosylated IgG.

Table S6. Identification of significant peak changes for ovalbumin.

**Significant peaks identified with PC1 [cm^{-1}]**	**Bond type**	**Significant peaks identified with PC2 [cm^{-1}]**	**Bond type**	**Peaks identified with PC2 [cm^{-1}]**	**Bond type**
**900-915**	Pro,Val, Ser[a]	480-495	S-S	1390	Amide III (α)
**930-940**	N-Cα-C	550	S-S	1415	Ser, Pro[a]
**1015-1020**	Phe	630	Tyr	1460	C-H
**1200**	Amide II	790-795	Glu, Pro[a]	1485-1495	C-H (α/β)
**1750 -1860**	C=O	820-830	Tyr (β)	1510	Indole ring (Trp)
		840	Tyr (α/β)	1530-1540	Amide II
		945	N-Ca-C	1550	Amide II
		1060	Arg, His, Val, Glu[a]	1560	Amide II
		1090	His, Ser, Glu[a]	1720-1730	C=O, protonated Glu [3]
		1150-1160	Phe, Trp[a]	1760	?
		1165	Phe, Trp[a]	1780	?
		1180-1190	Phe, Trp[a]	1800	?
		1235	Amide III (β)	1810	?
		1360	Trp, Ca-Hα	1850	C=O

(a) According to De Gelder et al. (2007) [2]. (b) Likely changes in the backbone (N-Cα-C).

Table S7. Identification of significant peak changes for *Lm*TIM$_{E65Q}$.

**Significant peaks identified with PC1 [cm^{-1}]**	**Bond type**	**Significant peaks identified with PC2 [cm^{-1}]**	**Bond type**	**Peaks identified with PC2 [cm^{-1}]**	**Bond type**
**980-1150**	Phe, C-N	560	S-S	1100	Phe
**1180**	Phe, Trp[a]	580	S-S	1130	C-N
**1205**	Amide II	645	Tyr	1165	Phe, Trp[a]
**1220-1240**	Amide III	660-680	Glu, Pro, His, Trp, Val[a]	1180-1190	Phe, Trp[a]
**1275**	Amide II	1000	Phe	1235	Amide III (β)
**1385**	Amide III (α)	1050	Phe	1250	Amide III
**1740-1820**	C=O	1080	Phe, Trp[a]	1260	Glu, Arg, Tyr, Pro[a]
		1090	Phe, Trp[a]	1360	Trp,Ca-Hα

(a) According to De Gelder et al. (2007) [2]. (b) Likely changes in the backbone (N-Cα-C).

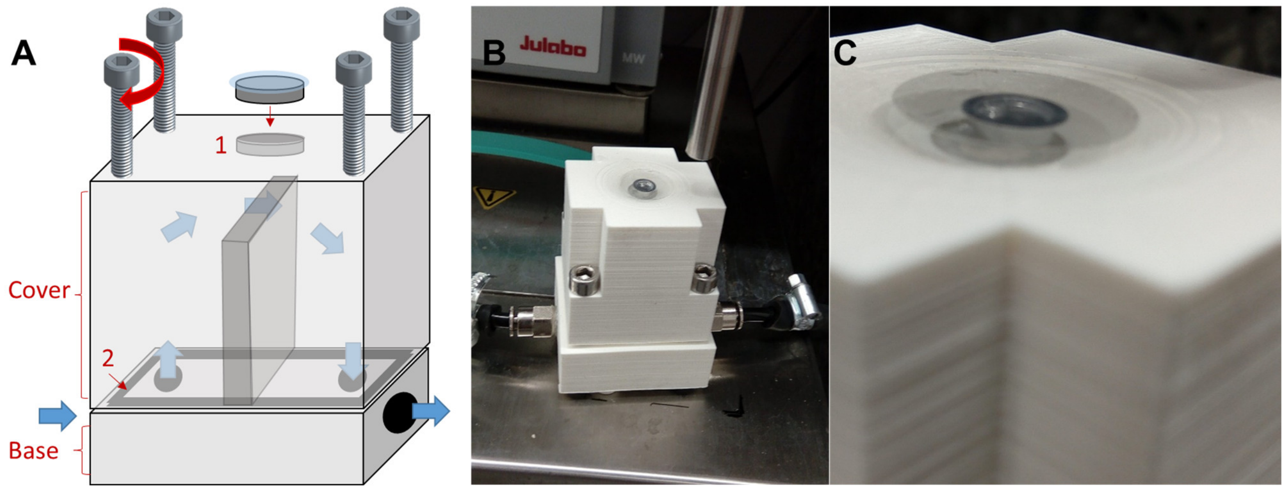

Figure S1. 3D-printed, heat exchange prototype for thermal ramping of protein solution samples for Raman spectroscopy sampling. (A) Schematic cartoon. 1: Sample holder, 2: rubber insert into groove. Blue arrows depicts the water flow, (B) final design, (C) close-up of the sample holder. An aluminum disk is in direct contact with the water, while an aluminum crusible is placed on it. The class cover-holder is sealed with vacume grease.

3.1 Alpha helical proteins

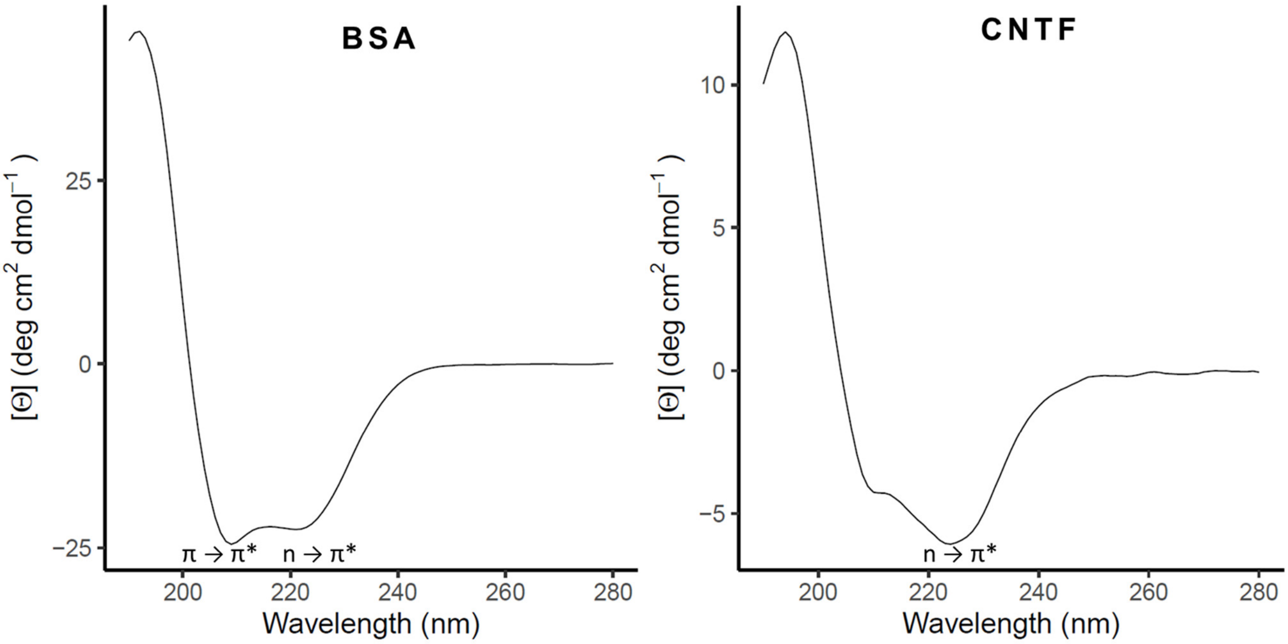

Figure S2. CD-spectra of BSA and CNTF before thermal denaturation.

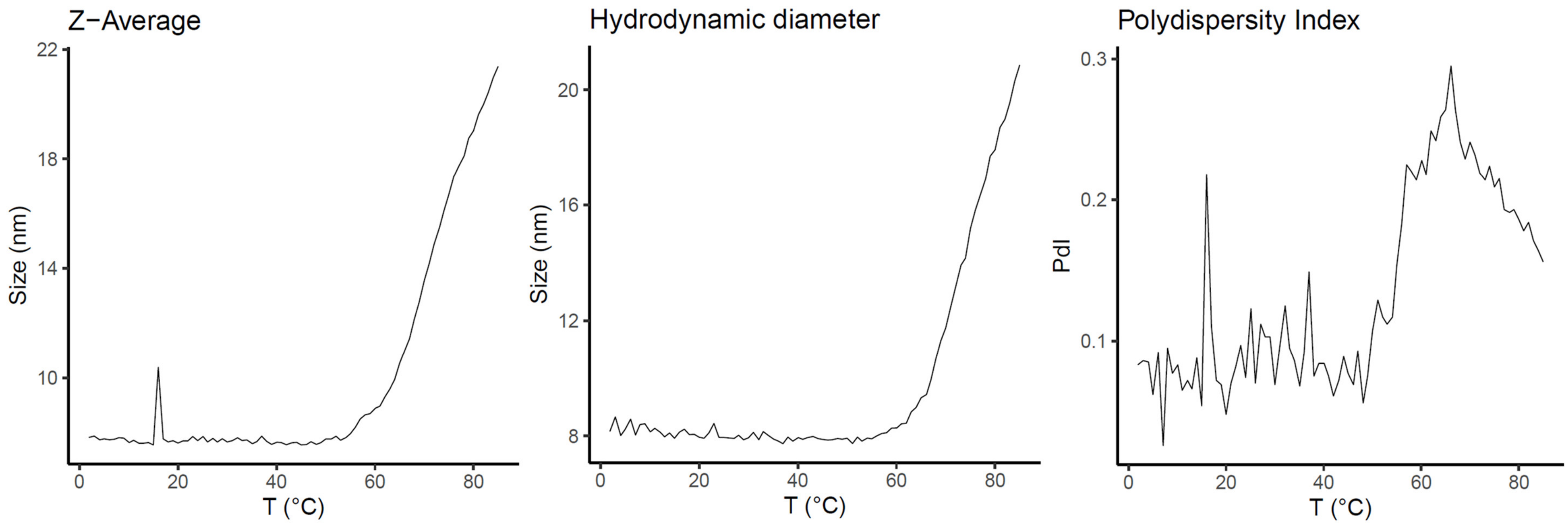

Figure S3. Dynamic light scattering thermal ramping profile of BSA. The DLS CNTF data was published recently [4]. The peak at 18 °C (Z-average and Polydispersity Index) is likely due to an inpurity or airbubble in the sample.

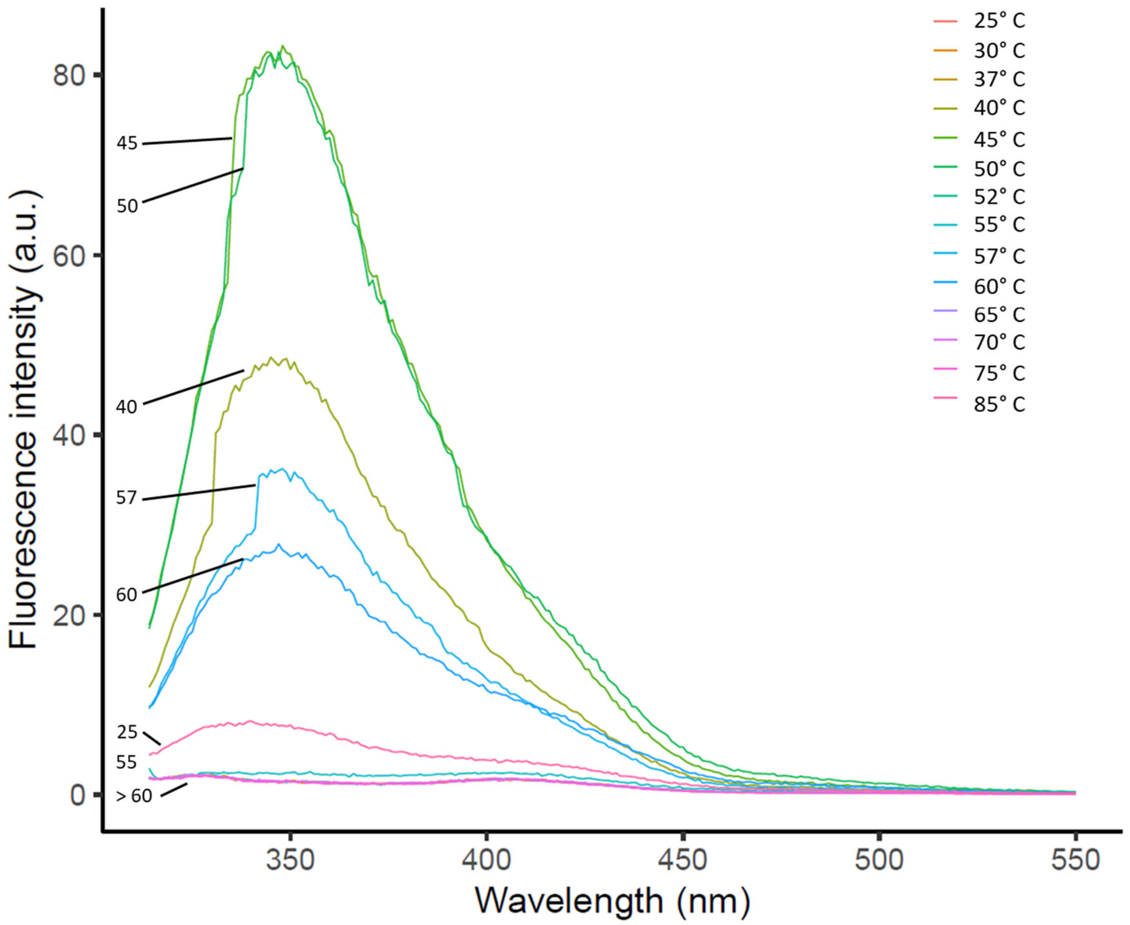
Fluorescence intensity (a.u.)
Wavelength (nm)
80
60
40
20
0
350
400
450
500
550
45
50
40
57
60
25
55
> 60
25° C
30° C
37° C
40° C
45° C
50° C
52° C
55° C
57° C
60° C
65° C
70° C
75° C
85° C

(A)

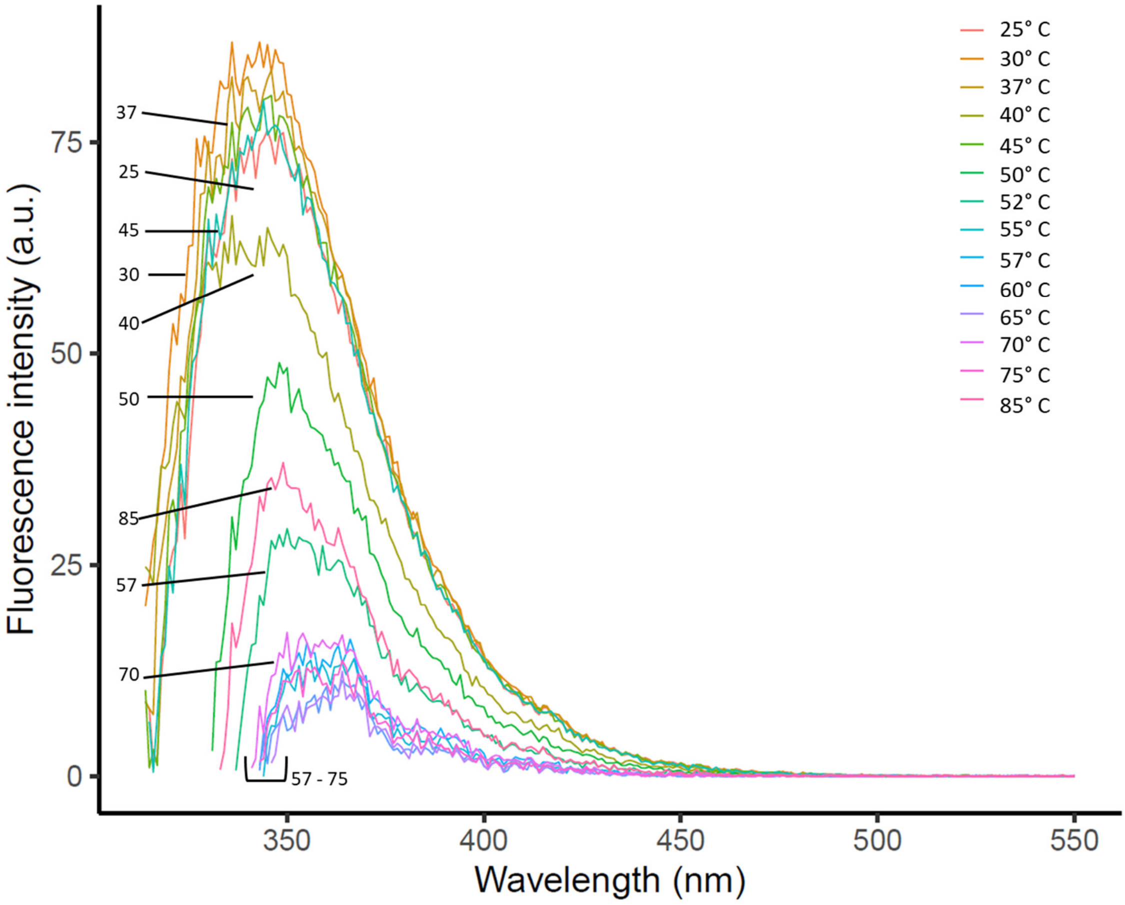

(B)

Figure S4. (**A**) Tryptophan fluoresence spectra of BSA upon thermal unfolding. (**B**) Tryptophan fluoresence spectra of CNTF upon thermal unfolding.

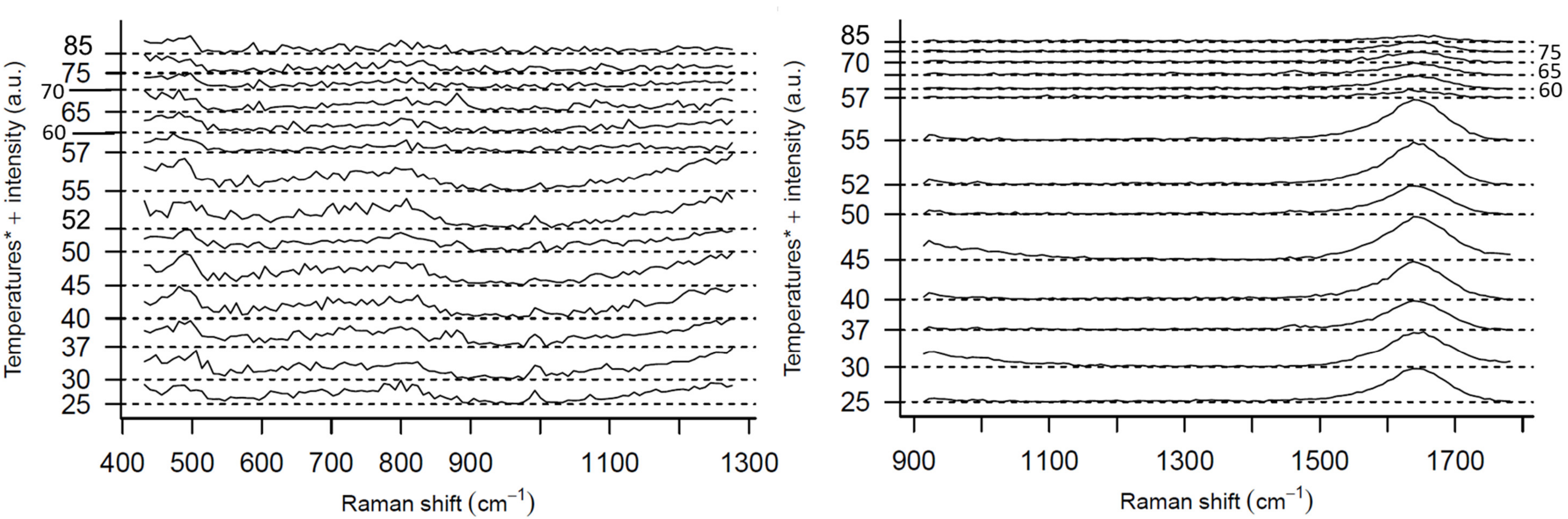

Figure S5. Non-normalized Time gated Raman spectra of BSA after thermal incubation: detector scan from 400 – 1300 cm^{-1} (left), detector scan from 900 – 1800 cm^{-1} (right). (*) The different spectra at different temperatures are stacked above each other for clarity and to compare the spectra in one representation.

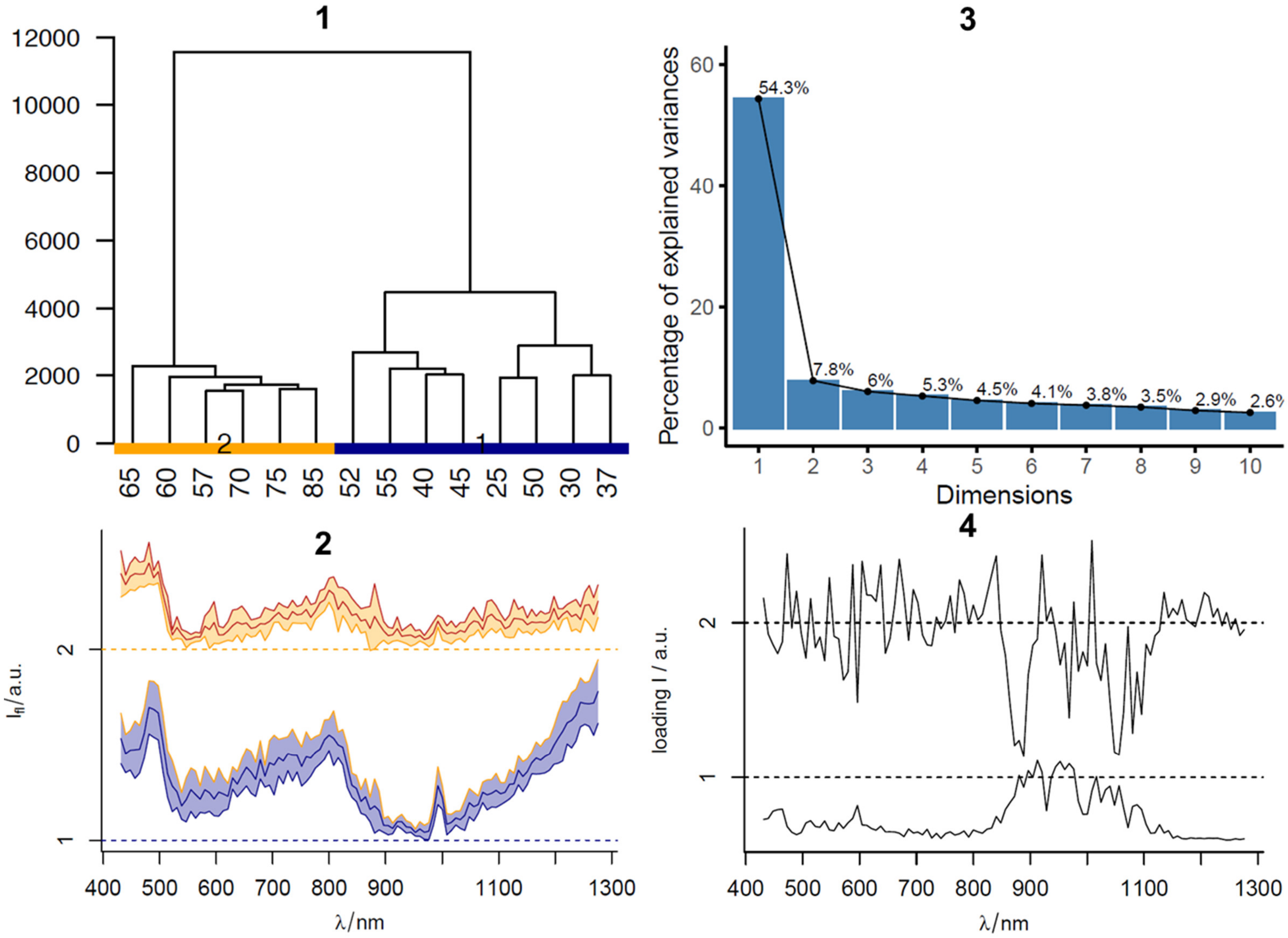
1
12000
10000
8000
6000
4000
2000
0
2
1
65 60 57 70 75 85 52 55 40 45 25 50 30 37
2
I_{fl}/a.u.
2
1
400 500 600 700 800 900 1100 1300
λ/nm
3
Percentage of explained variances
60
40
20
0
54.3%
7.8%
6%
5.3%
4.5%
4.1%
3.8%
3.5%
2.9%
2.6%
1 2 3 4 5 6 7 8 9 10
Dimensions
4
loading l / a.u.
2
1
400 500 600 700 800 900 1100 1300
λ/nm

(A)

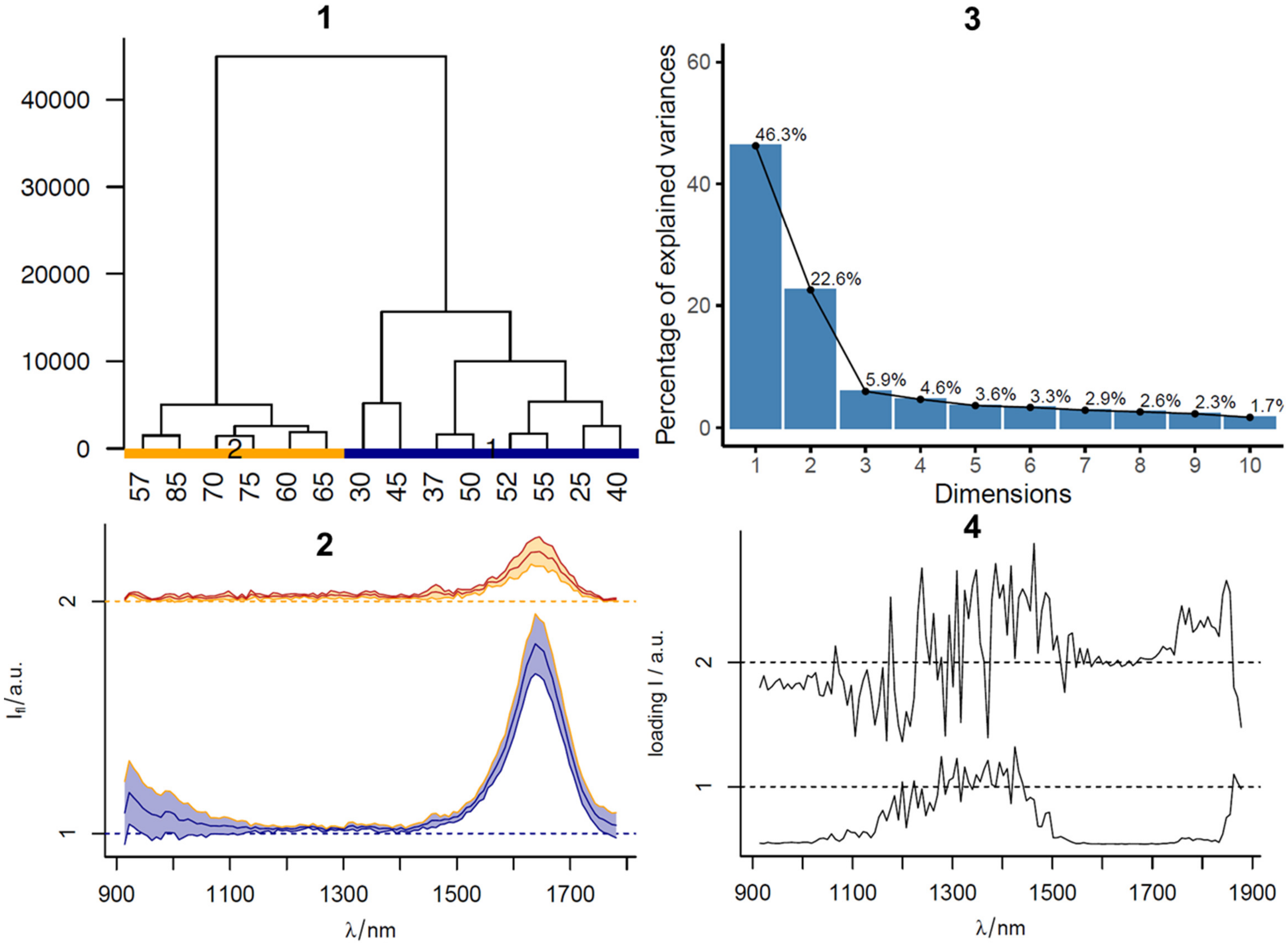

(B)

Figure S6. (**A**) BSA Hierarchical clustering: (1) dendrogram (2) cluster means and PCA: (3) Scree plot [5], and (4) loadings. (**B**) BSA Hierarchical clustering: (1) dendrogram (2) cluster means and PCA: (3) Scree plot, and (4) loadings.

3.2 Beta sheet proteins

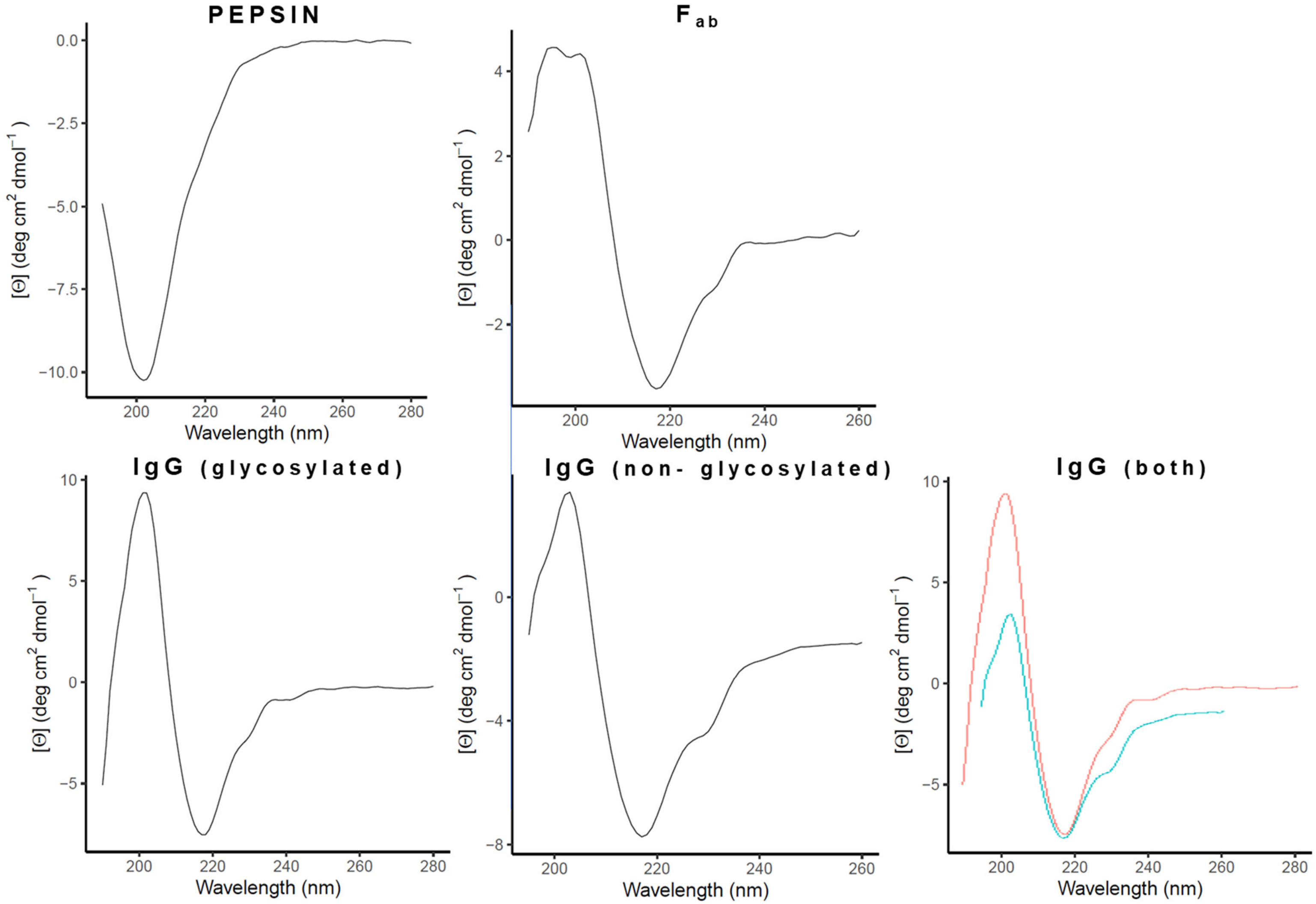

Figure S7. CD-spectra of pepsin, F_{ab} fragment, and IgG (glycosylated (left and red); non-glycosylated (middle and blue)) before thermal denaturation.

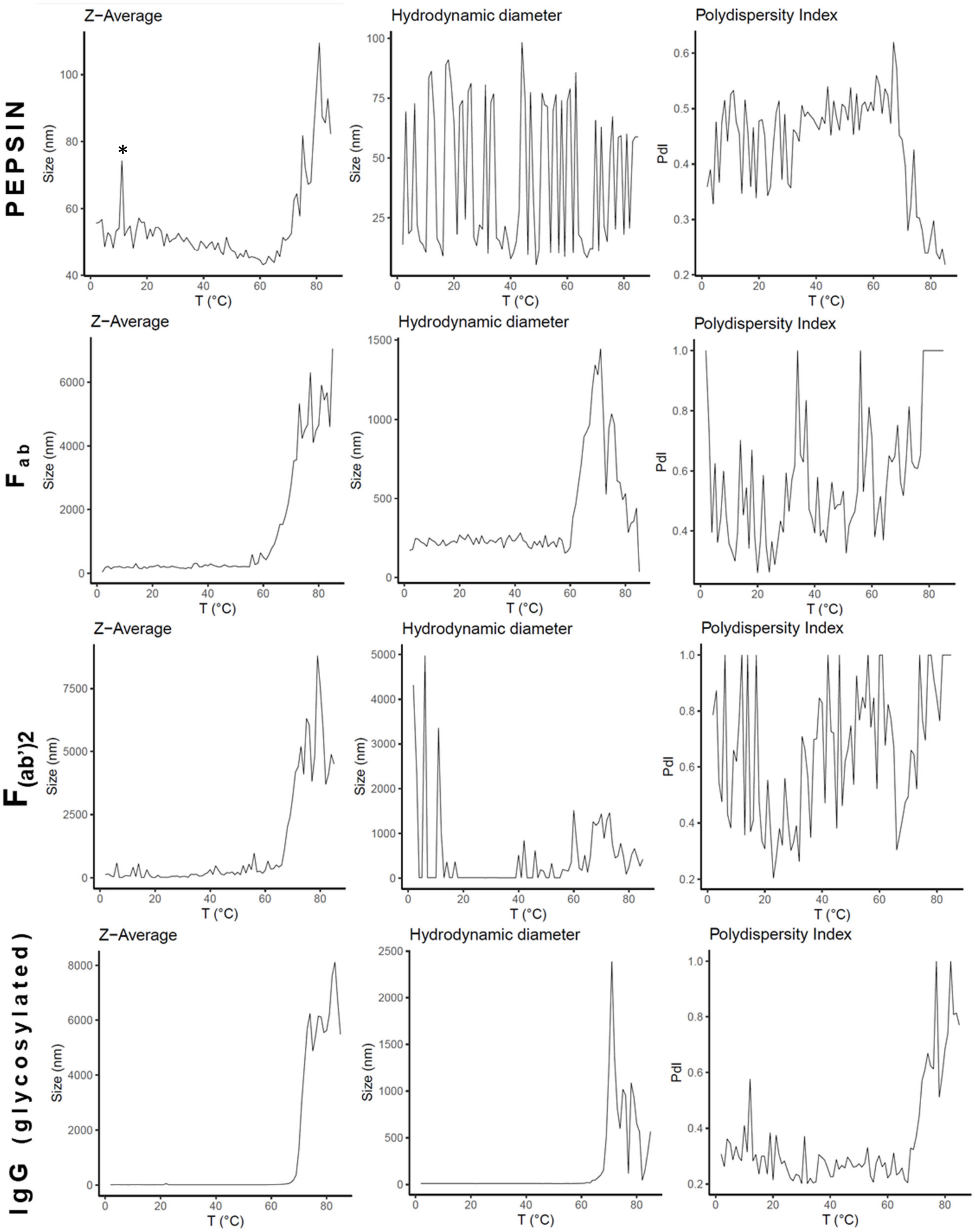

Figure S8. Dynamic light scattering thermal ramping profiles, from top to bottom, of Pepsin, F_{ab}, $F_{(ab')2}$, and IgG (glycosylated). (*) The peak at 15 °C (Z-average) is an inpurity or airbubble in the sample.

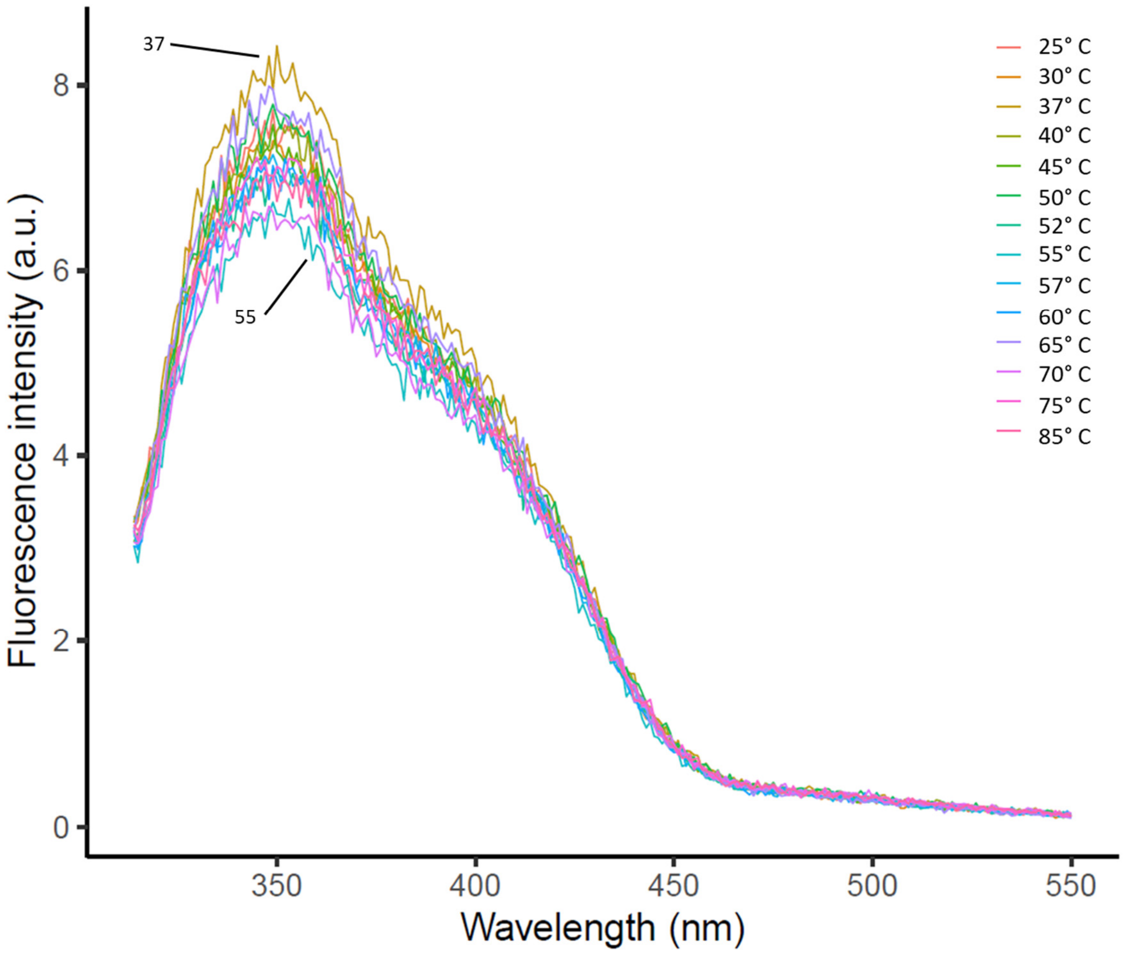

37
55
Fluorescence intensity (a.u.)
Wavelength (nm)
0
2
4
6
8
350
400
450
500
550
25° C
30° C
37° C
40° C
45° C
50° C
52° C
55° C
57° C
60° C
65° C
70° C
75° C
85° C

(**A**)

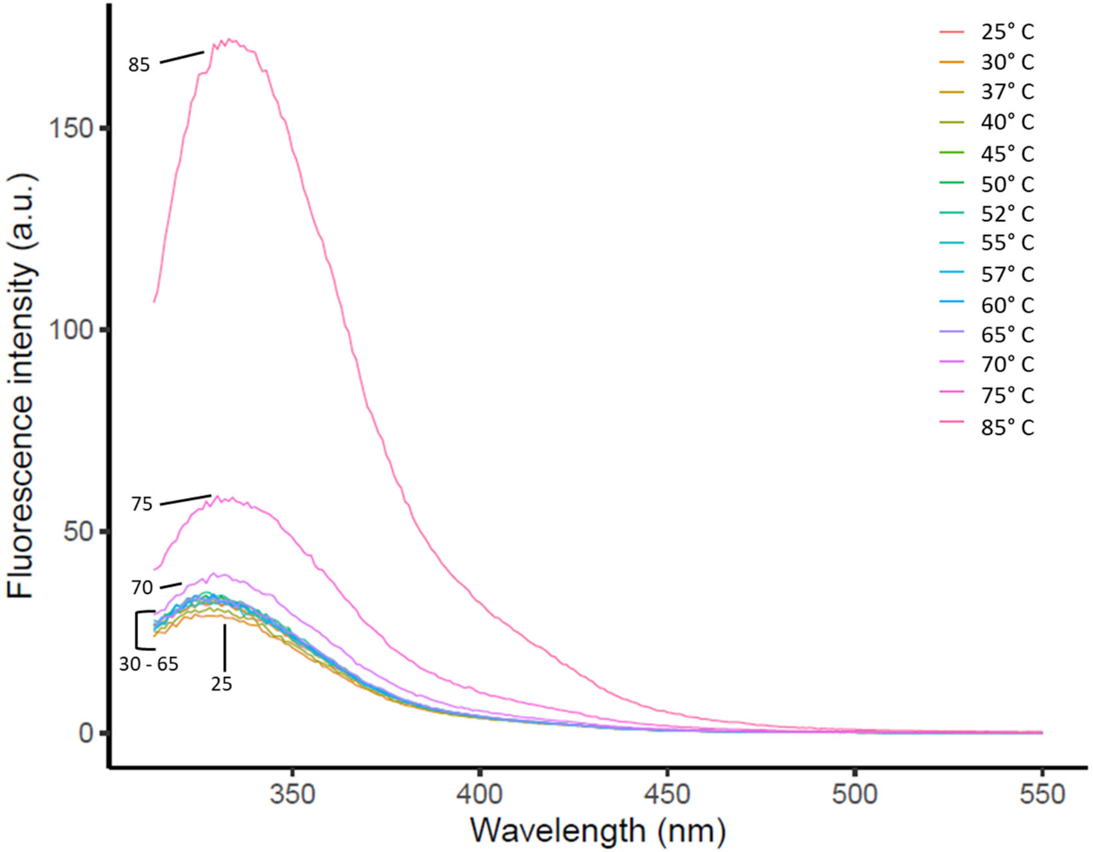

Fluorescence intensity (a.u.)
Wavelength (nm)
0
50
100
150
350
400
450
500
550
85
75
70
30 - 65
25
25° C
30° C
37° C
40° C
45° C
50° C
52° C
55° C
57° C
60° C
65° C
70° C
75° C
85° C

(**B**)

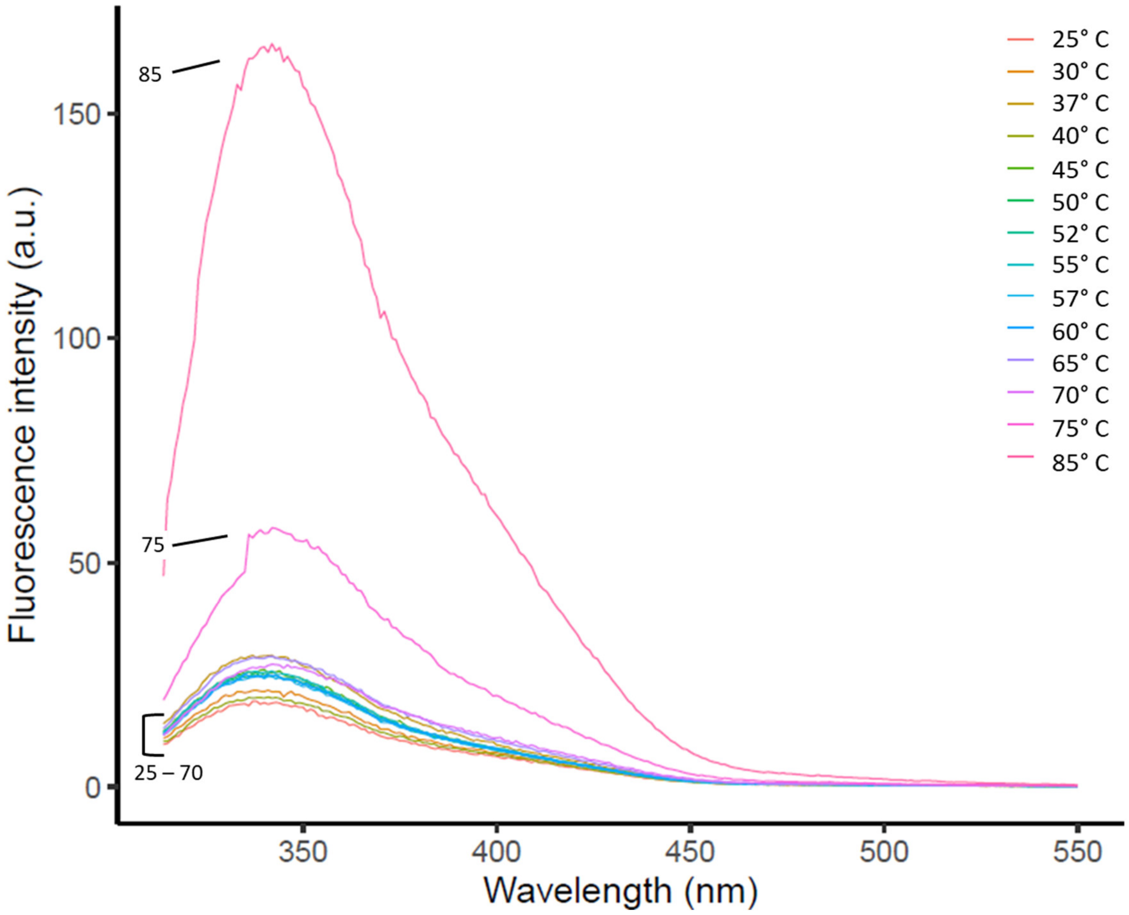

(C)

Figure S9. (**A**) Tryptophan fluorescence spectra of pepsin upon thermal unfolding. (**B**) Tryptophan fluorescence spectra of F_{ab} upon thermal unfolding. (**C**) Tryptophan fluorescence spectra of IgG (glycosylated) upon thermal unfolding.

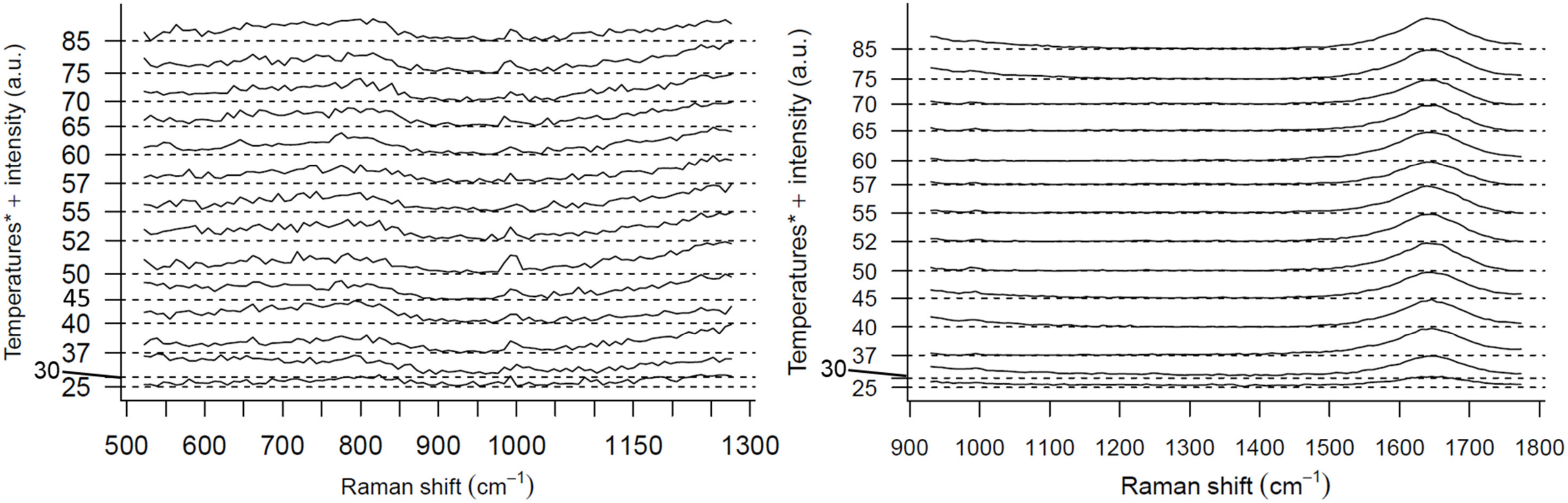

Figure S10. Non-normalized time-gated Raman spectra of pepsin after thermal incubation; left panel: detector scan from 500 – 1300 cm^{-1} (line at 880 cm^{-1}), right panel: detector scan from 900 – 1800 cm^{-1}. (*) The different spectra at different temperatures are stacked above each other for clarity and to compare the spectra in one representation.

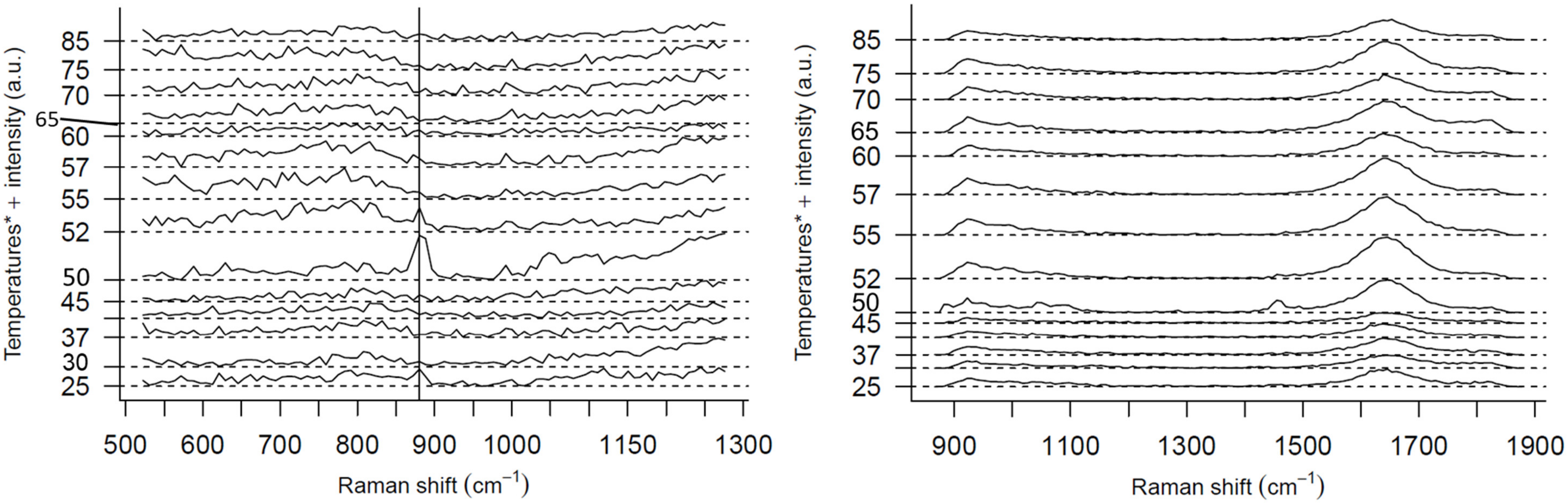

Figure S11. Non-normalized time-gated Raman spectra of IgG (glycosylated) after thermal incubation; left panel: detector scan from 400 – 1300 cm^{-1} (line at 880 cm^{-1}), right panel: detector scan from 900 – 1900 cm^{-1}. (*) The different spectra at different temperatures are stacked above each other for clarity and to compare the spectra in one representation.

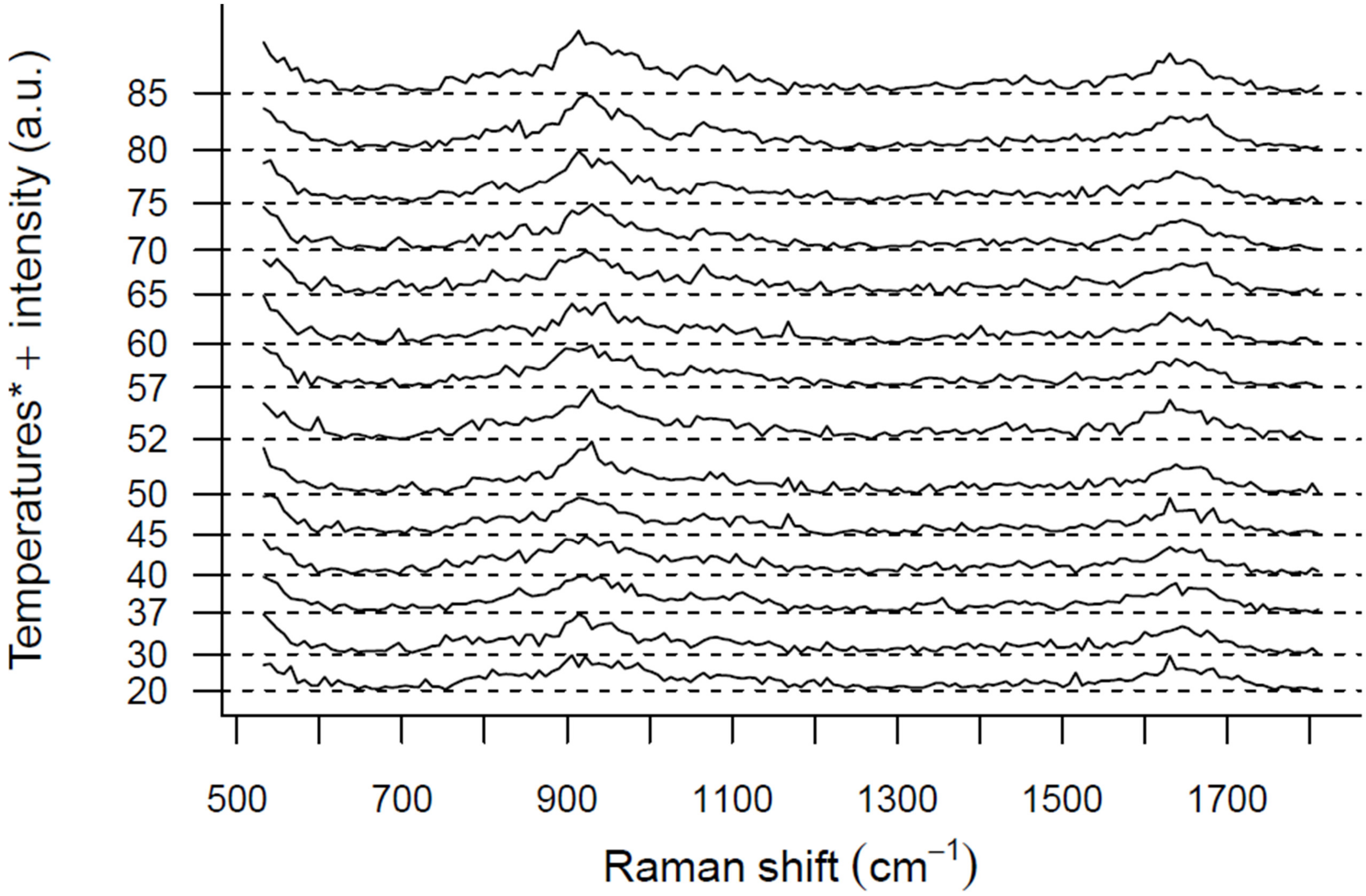

Figure S12. Non-normalized time-gated Raman spectra of IgG (non-glycosylated) after continuous thermal incubation. (*) The different spectra at different temperatures are stacked above each other for clarity and to compare the spectra in one representation.

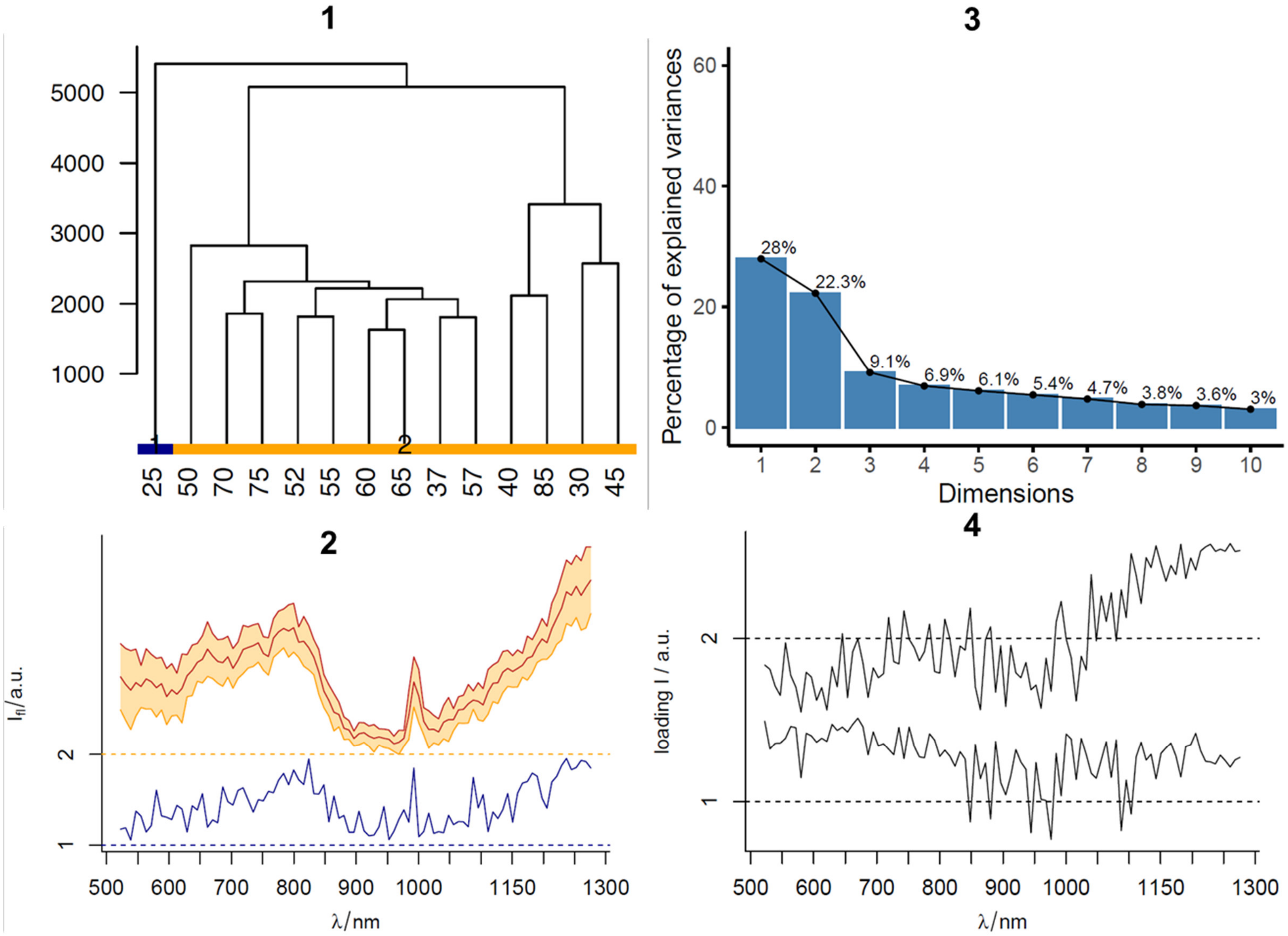
1
5000
4000
3000
2000
1000
1
2
25 50 70 75 52 55 60 65 37 57 40 85 30 45
3
Percentage of explained variances
60
40
20
0
28%
22.3%
9.1%
6.9%
6.1%
5.4%
4.7%
3.8%
3.6%
3%
1 2 3 4 5 6 7 8 9 10
Dimensions
2
I_{fl}/a.u.
2
1
500 600 700 800 900 1000 1150 1300
λ/nm
4
loading I / a.u.
2
1
500 600 700 800 900 1000 1150 1300
λ/nm

(**A**)

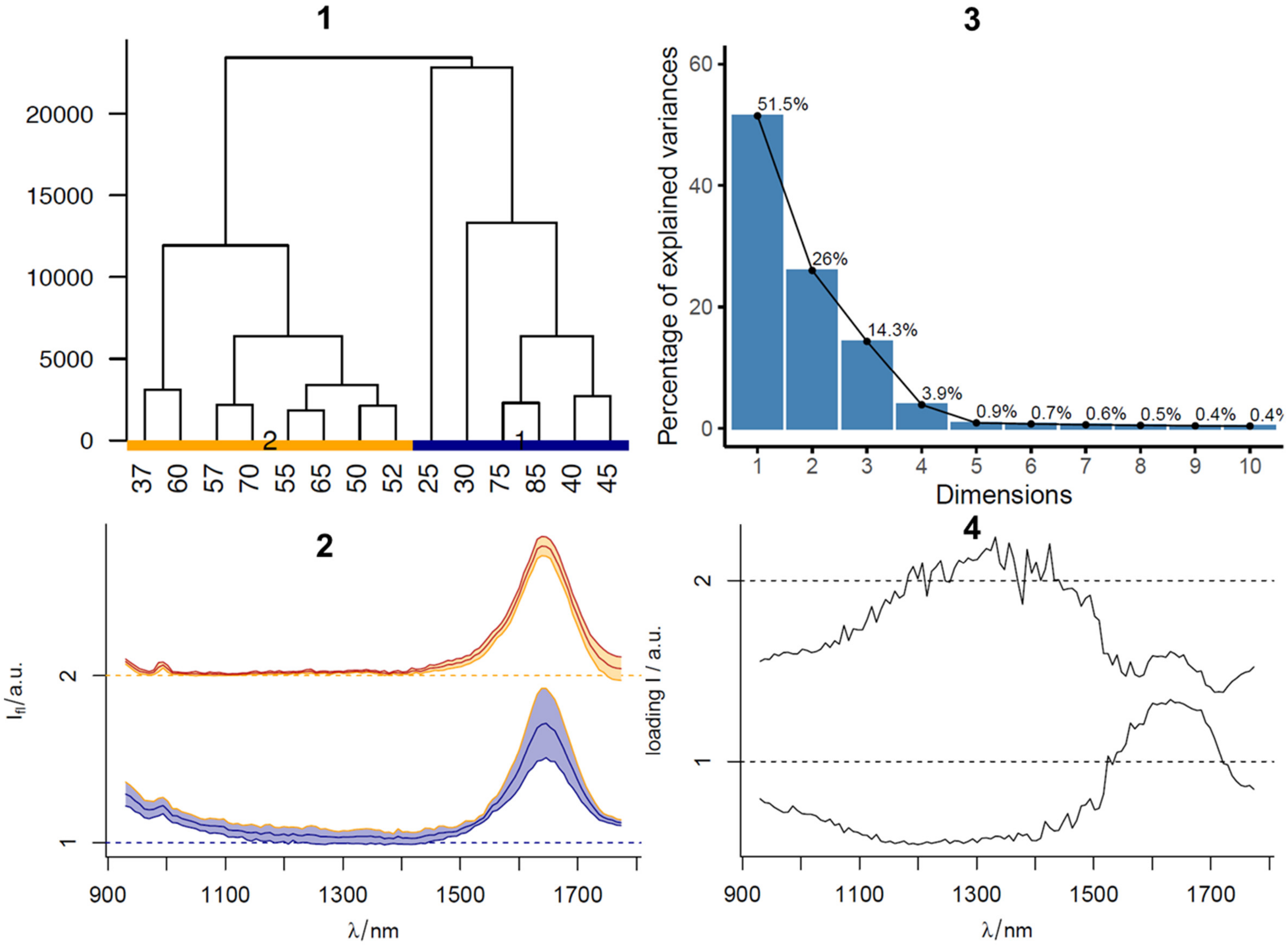

(B)

Figure S13. (**A**) Pepsin hierarchical clustering: (1) dendrogram (2) cluster means and PCA: (3) Scree plot, and (4) loadings. (**B**) Pepsin hierarchical clustering: (1) dendrogram (2) cluster means and PCA: (3) Scree plot, and (4) loadings.

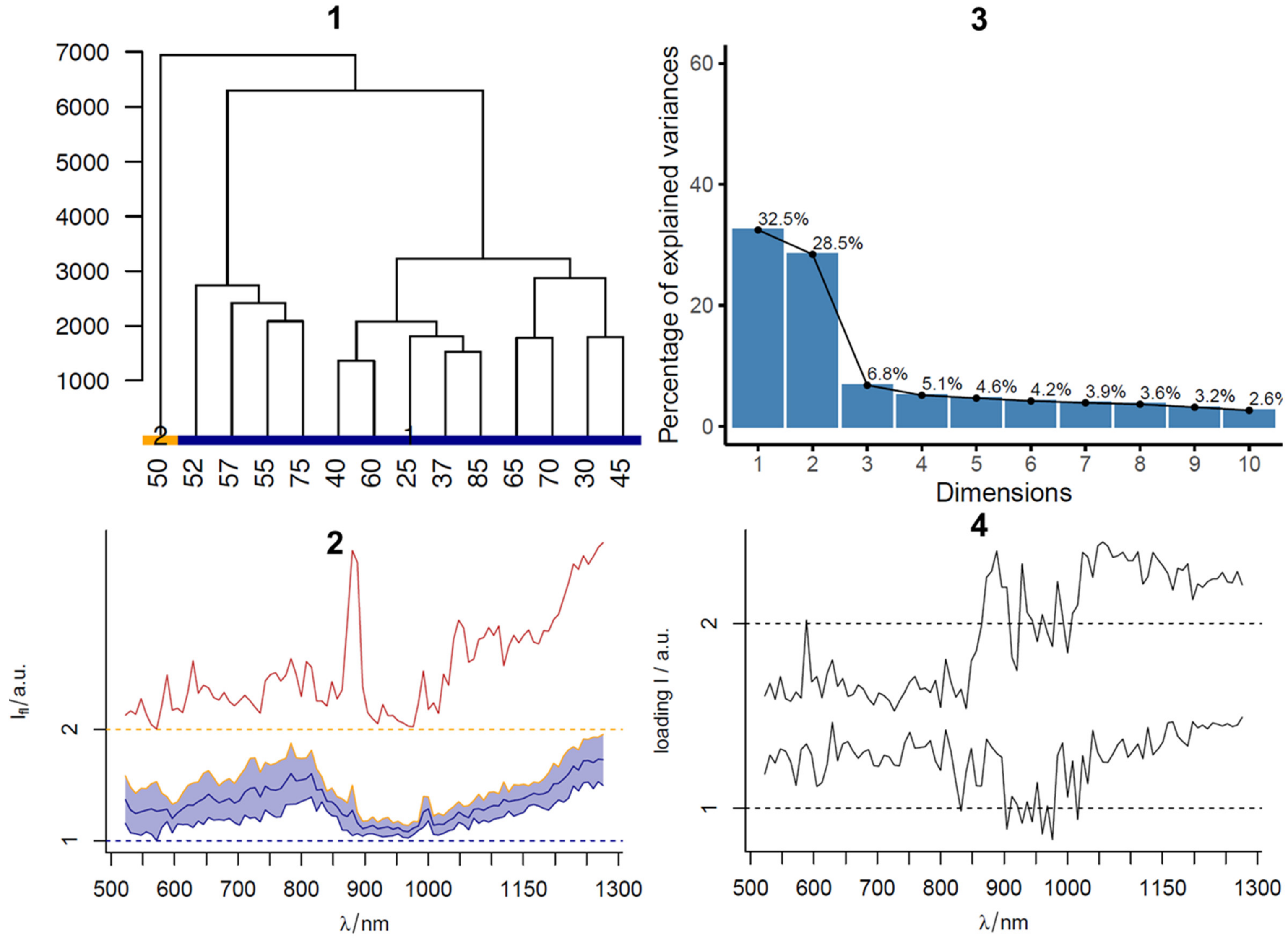
1
7000
6000
5000
4000
3000
2000
1000
2
1
50
52
57
55
75
40
60
25
37
85
65
70
30
45
3
Percentage of explained variances
60
40
20
0
32.5%
28.5%
6.8%
5.1%
4.6%
4.2%
3.9%
3.6%
3.2%
2.6%
1
2
3
4
5
6
7
8
9
10
Dimensions
2
I_{fl}/a.u.
2
1
500
600
700
800
900
1000
1150
1300
λ/nm
4
loading I / a.u.
2
1
500
600
700
800
900
1000
1150
1300
λ/nm

(**A**)

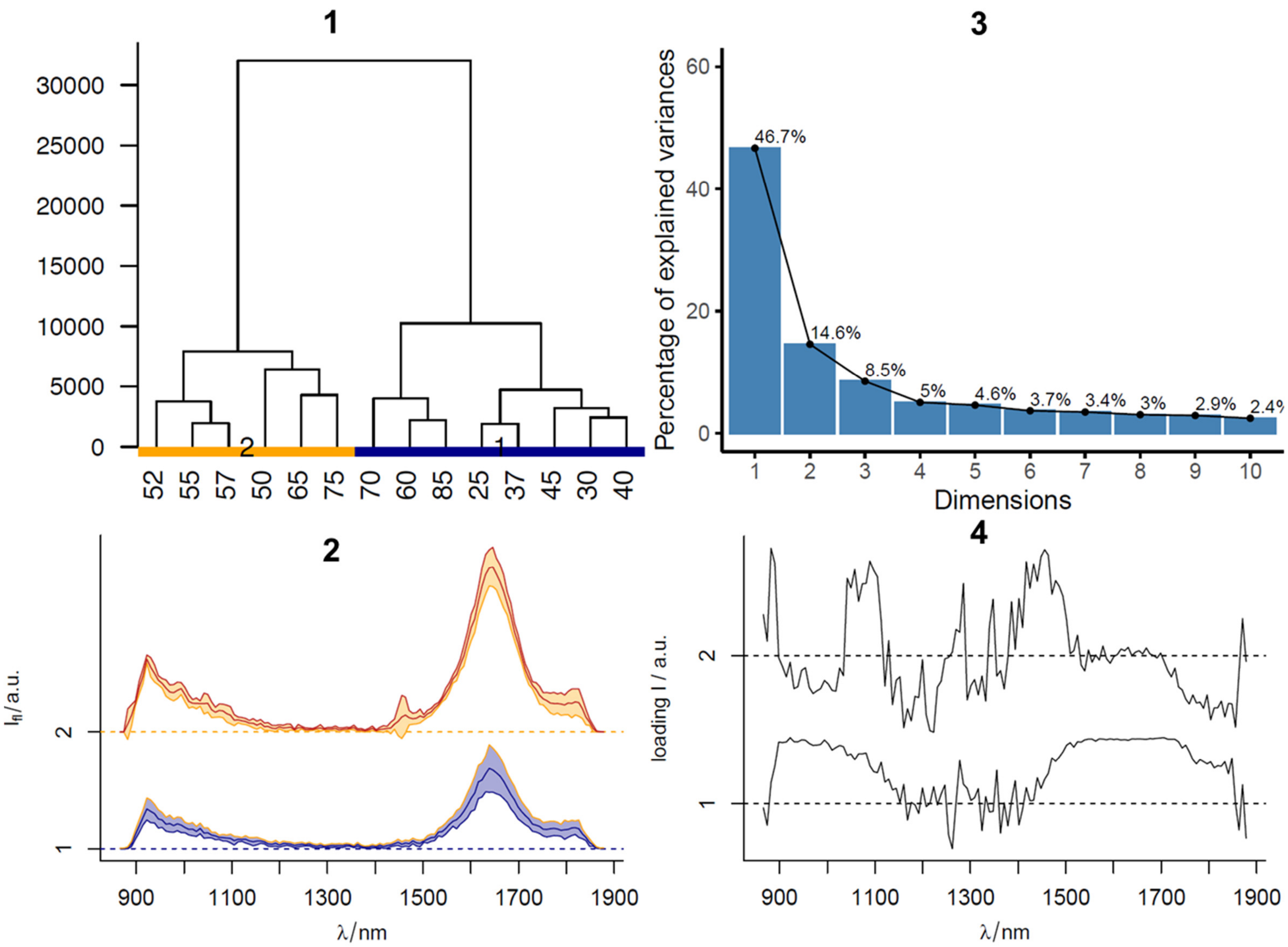

(**B**)

Figure S14. (**A**) IgG (glycosylated) Hierarchical clustering: (1) dendrogram (2) cluster means and PCA: (3) Scree plot, and (4) loadings. (**B**) IgG (glycosylated) Hierarchical clustering: (1) dendrogram (2) cluster means and PCA: (3) Scree plot, and (4) loadings.

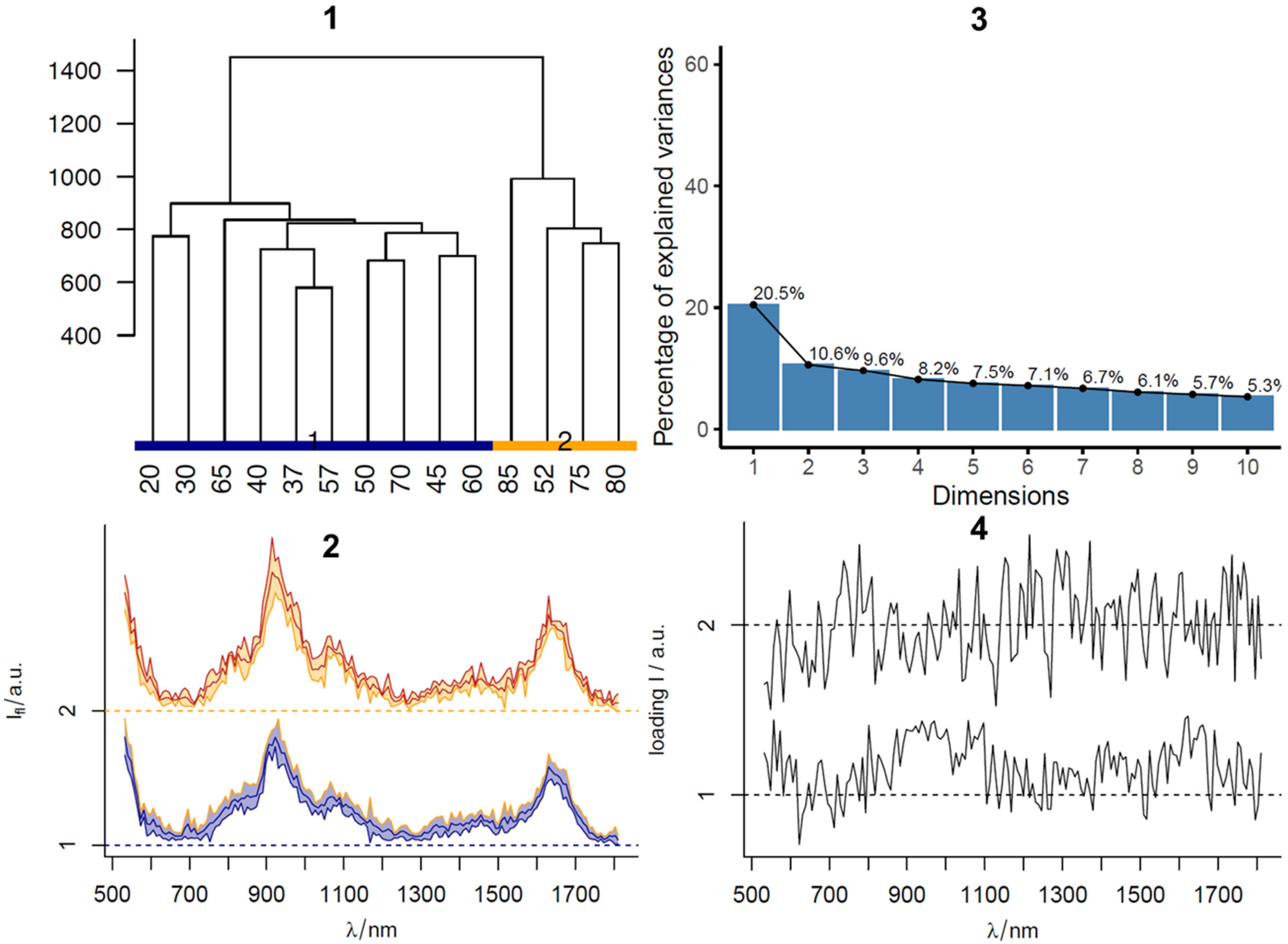

Figure S15. IgG (non-glycosylated) Hierarchical clustering: (1) dendrogram (2) cluster means and PCA: (3) Scree plot, and (4) loadings.

3.3 Alha helical / Beta sheet proteins

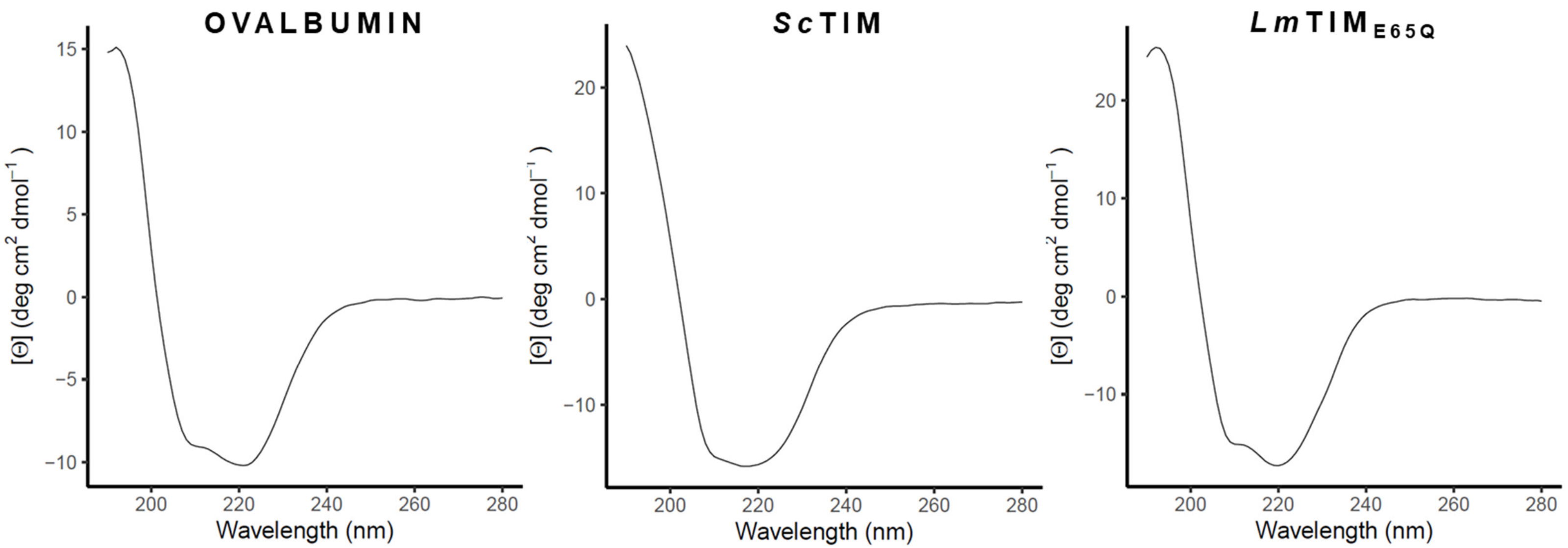

Figure S16. CD-spectra of ovalbumin, and triosephosphate isomerase (*Saccharomyces cerevisiae* and *Leishmania mexicana* E65Q) before thermal denaturation.

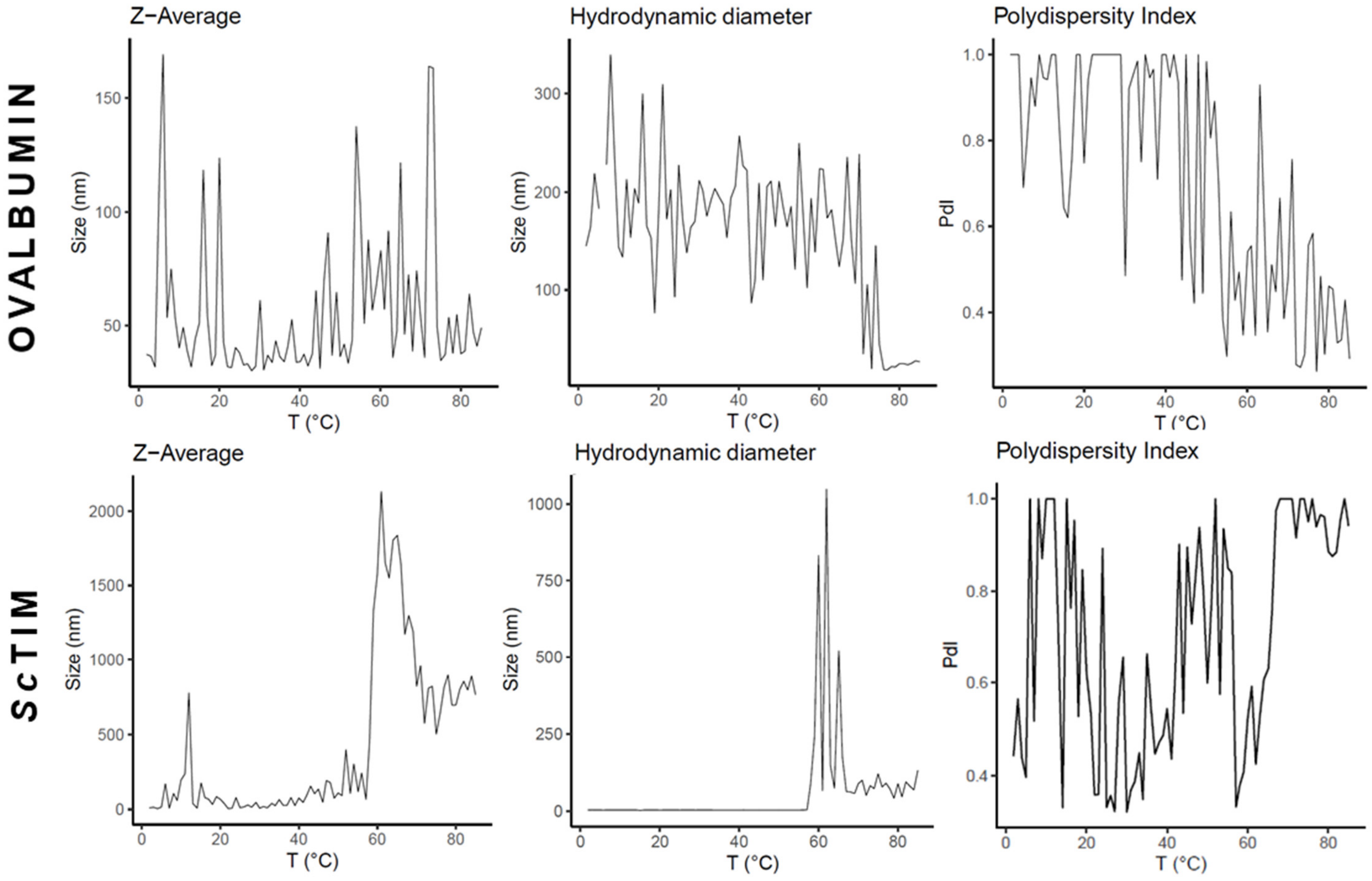

Figure S17. Dynamic light scattering thermal ramping profiles of ovalbumin and *Sc*TIM.

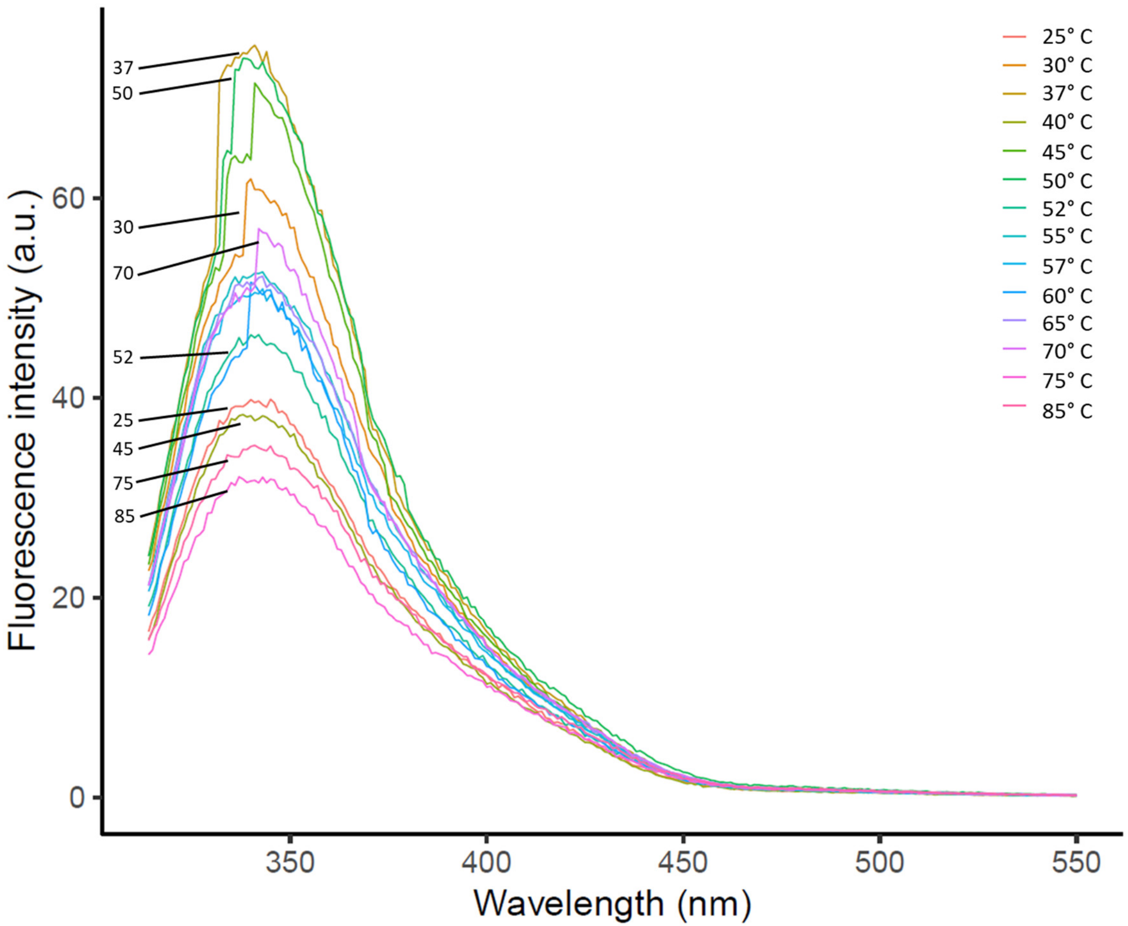
Fluorescence intensity (a.u.)
Wavelength (nm)
0
20
40
60
350
400
450
500
550
37
50
30
70
52
25
45
75
85
25° C
30° C
37° C
40° C
45° C
50° C
52° C
55° C
57° C
60° C
65° C
70° C
75° C
85° C

(**A**)

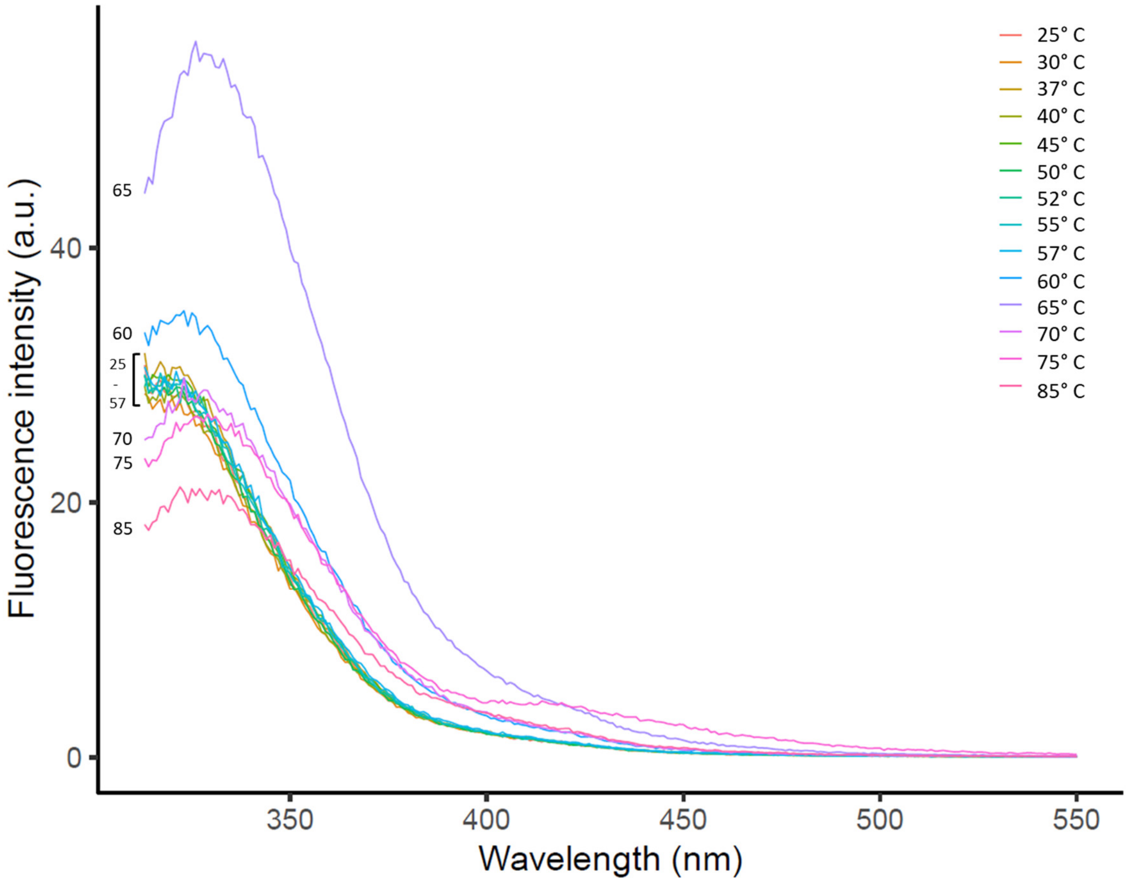
Fluorescence intensity (a.u.)
Wavelength (nm)
0
20
40
350
400
450
500
550
65
60
25
-
57
70
75
85
25° C
30° C
37° C
40° C
45° C
50° C
52° C
55° C
57° C
60° C
65° C
70° C
75° C
85° C

(**B**)

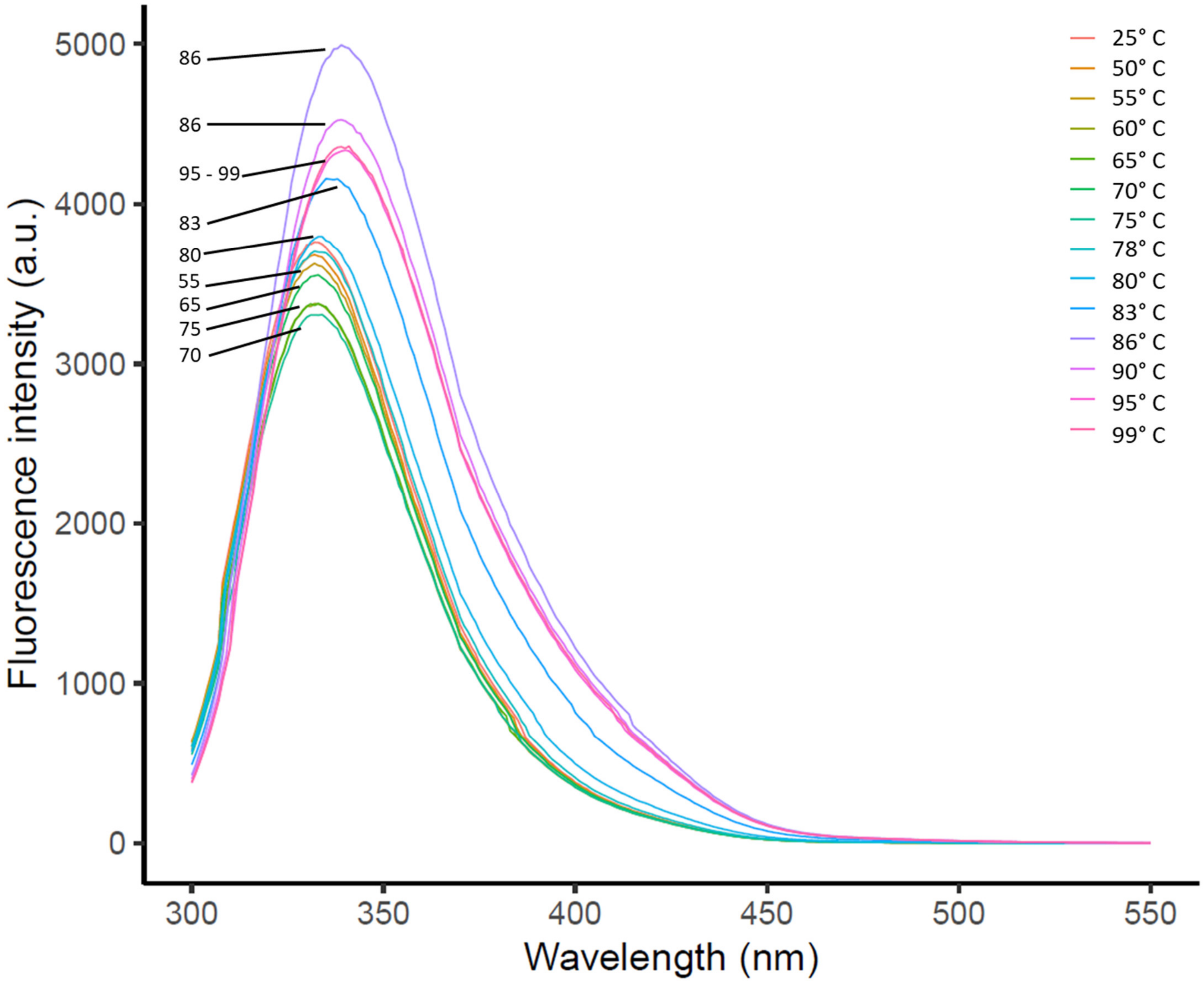

(**C**)

Figure S18. (**A**). Tryptophan fluoresence spectra of ovalbumin upon thermal unfolding. (**B**) Tryptophan fluoresence spectra of *Sc*TIM upon thermal unfolding. (**C**) Tryptophan fluoresence spectra of *Lm*TIM$_{E65Q}$ upon thermal unfolding.

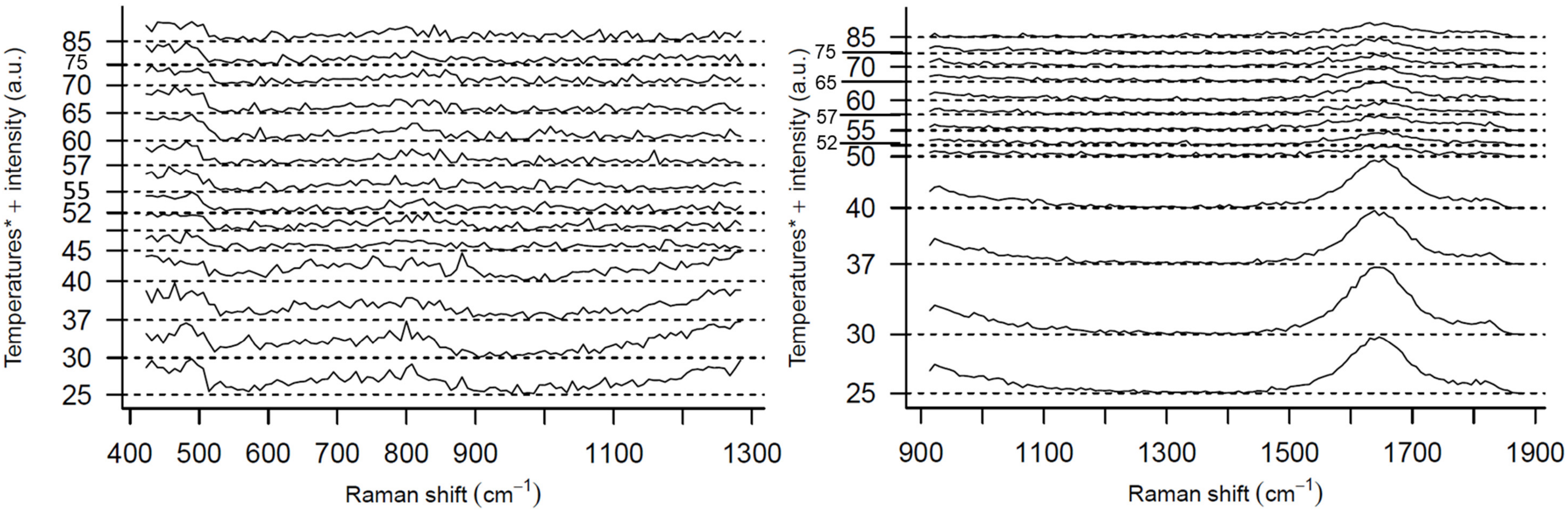

Figure S19. Non-normalized time-gated Raman spectra of ovalbumin after thermal incubation; left panel: detector scan from 400 – 1300 cm^{-1}, right panel: detector scan from 900 – 1900 cm^{-1}. (*) The different spectra at different temperatures are stacked above each other for clarity and to compare the spectra in one representation.

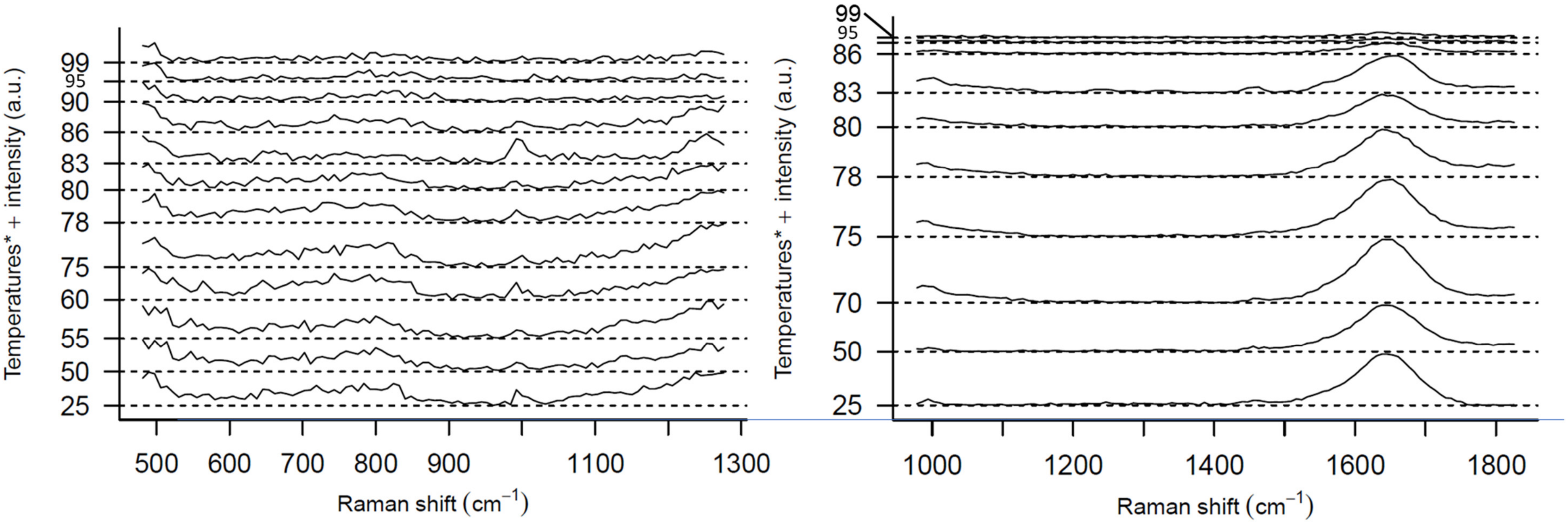

Figure S20. Non-normalized time-gated Raman spectra of *Lm*E65Q triosephosphate isomerase after thermal incubation; left panel: detector scan from 450 – 1300 cm^{-1}, right panel: detector scan from 900 – 1850 cm^{-1}. (*) The different spectra at different temperatures are stacked above each other for clarity and to compare the spectra in one representation.

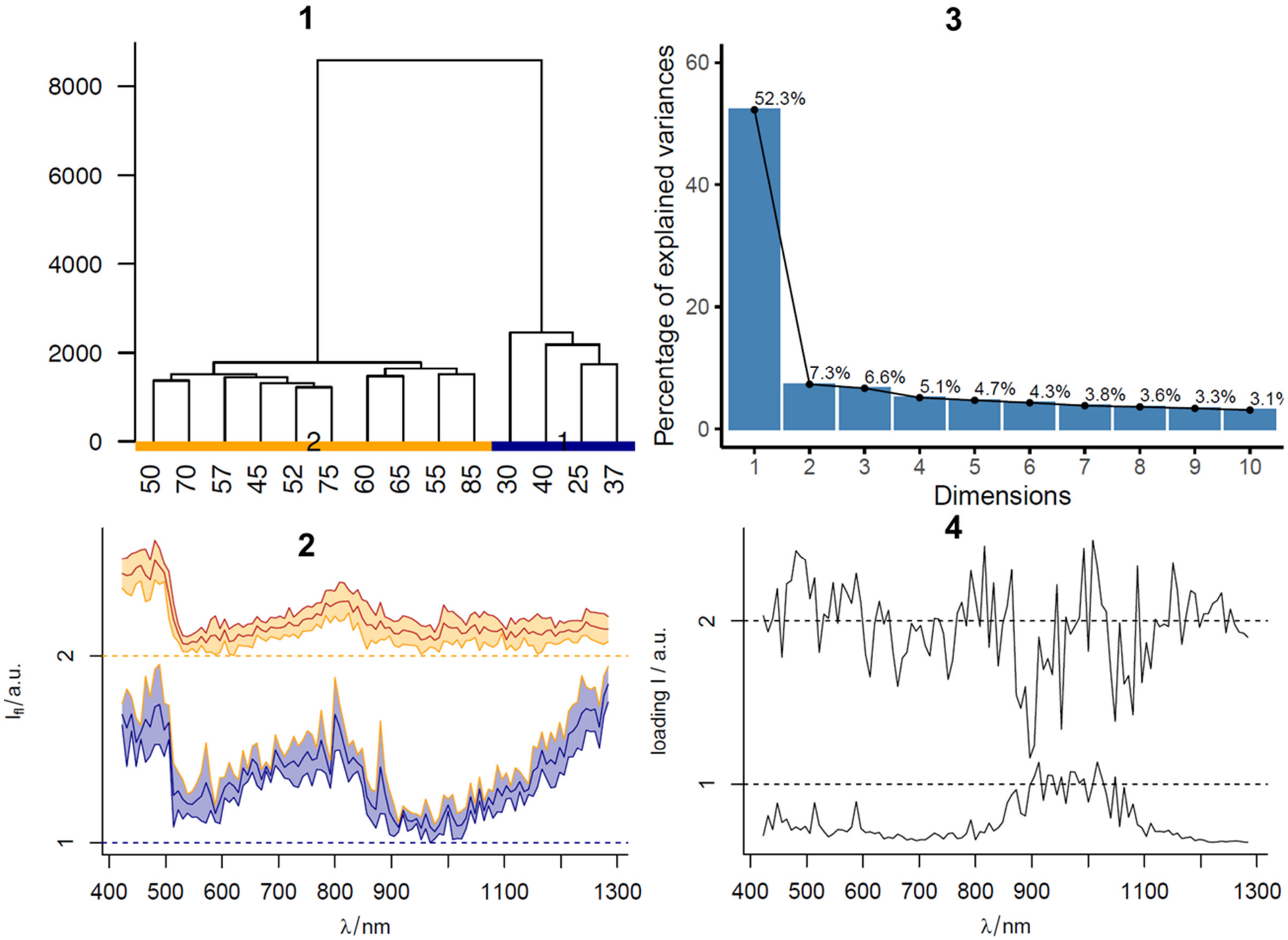

1
8000
6000
4000
2000
0
2
1
50 70 57 45 52 75 60 65 55 85 30 40 25 37
3
Percentage of explained variances
60
40
20
0
52.3%
7.3%
6.6%
5.1%
4.7%
4.3%
3.8%
3.6%
3.3%
1 2 3 4 5 6 7 8 9 10
Dimensions
2
I/a.u.
400 500 600 700 800 900 1100 1300
λ/nm
4
loading I / a.u.
400 500 600 700 800 900 1100 1300
λ/nm

(**A**)

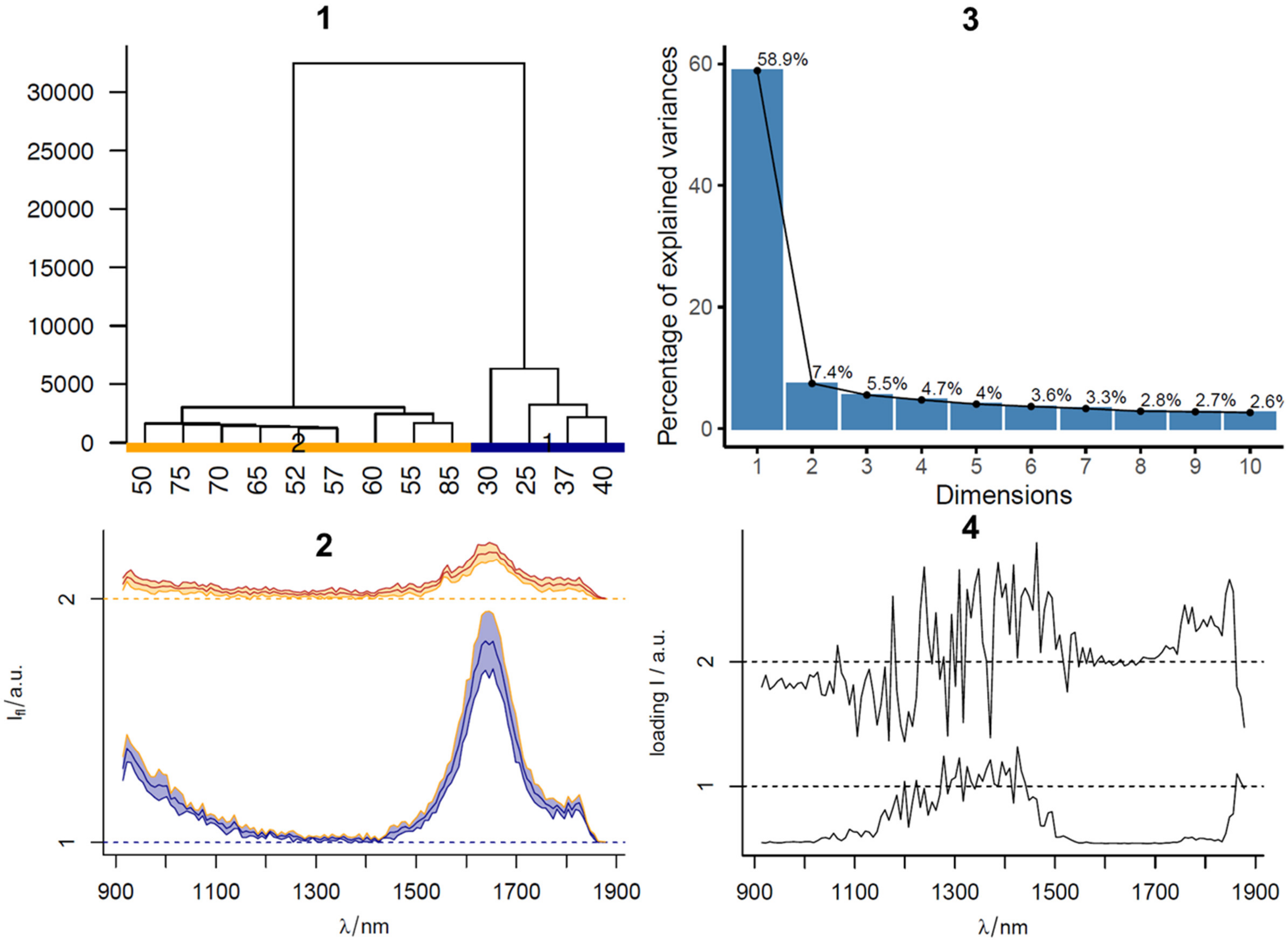

(**B**)

Figure S21. (**A**) Ovalbumin hierarchical clustering: (1) dendrogram (2) cluster means and PCA: (3) Scree plot, and (4) loadings. (**B**) Ovalbumin hierarchical clustering: (1) dendrogram (2) cluster means and PCA: (3) Scree plot, and (4) loadings.

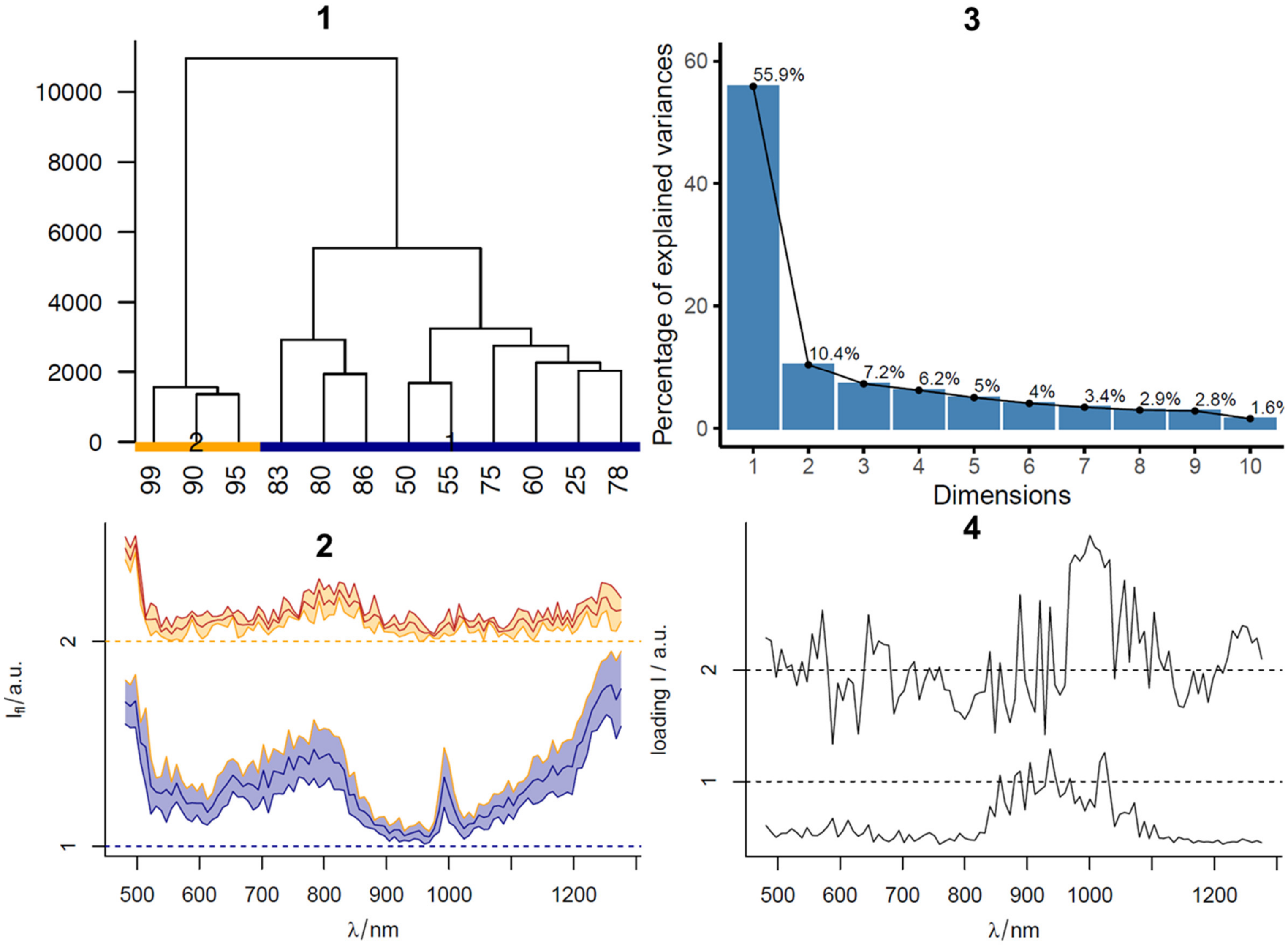
1
10000
8000
6000
4000
2000
0
2
1
99 90 95 83 80 86 50 55 75 60 25 78
3
Percentage of explained variances
60
40
20
0
55.9%
10.4%
7.2%
6.2%
5%
4%
3.4%
2.9%
2.8%
1 2 3 4 5 6 7 8 9 10
Dimensions
2
I_fl/a.u.
2
1
500 600 700 800 900 1000 1200
λ/nm
4
loading I / a.u.
2
1
500 600 700 800 900 1000 1200
λ/nm

(**A**)

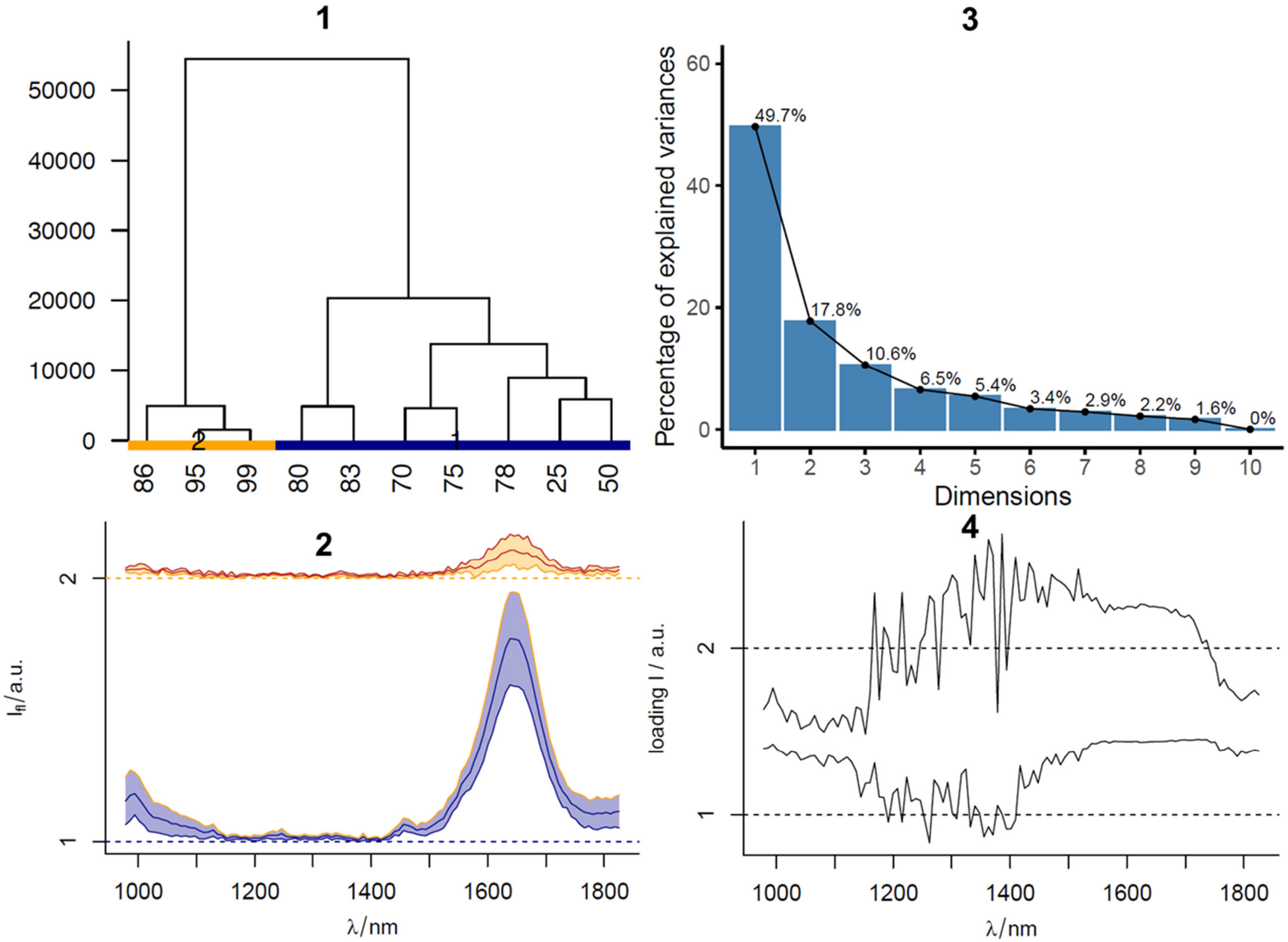

(**B**)

Figure S22. (**A**) *Lm*E65Q TIM hierarchical clustering: (1) dendrogram (2) cluster means and PCA: (3) Scree plot, and (4) loadings. (**B**) *Lm*E65Q TIM hierarchical clustering: (1) dendrogram (2) cluster means and PCA: (3) Scree plot, and (4) loadings

3.4. Time-gated Raman combined

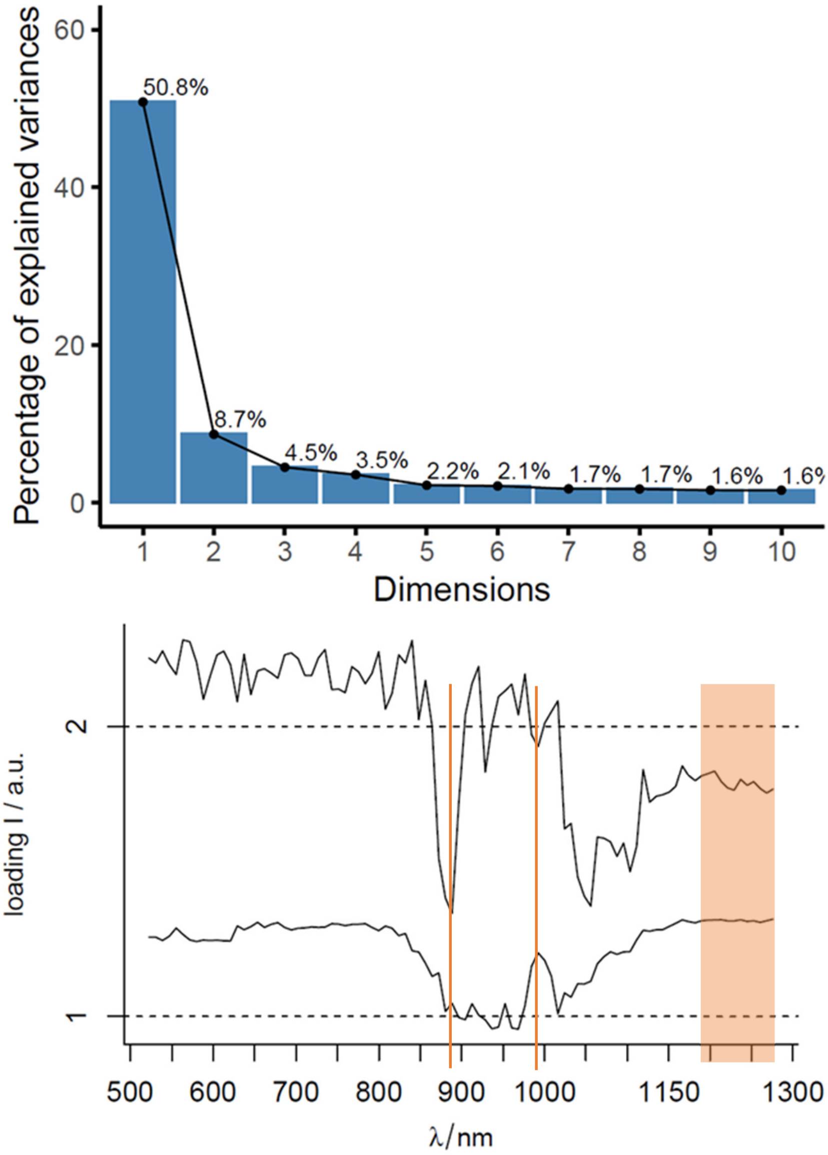

Figure S23. Time-gated Raman spectra hierarchical clustering of all spectra (500 – 1300 cm^{-1}); top: Scree plot, below loadings. Lines at 880 cm^{-1} (Trp) and ~ 1000 cm^{-1} (Phe). The orange region represents the amide III region.

References

1. Rygula, A.; Majzner, K.; Marzec, K.M.; Kaczor, A.; Pilarczyk, M.; Baranska, M. Raman spectroscopy of proteins: a review. *Journal of Raman Spectroscopy* **2013**, *44*, 1061-1076, doi:10.1002/jrs.4335.
2. De Gelder, J.; De Gussem, K.; Vandenabeele, P.; Moens, L. Reference database of Raman spectra of biological molecules. *Journal of Raman Spectroscopy* **2007**, *38*, 1133-1147, doi:10.1002/jrs.1734.
3. Gremlich, H.-U.B.Y. *Infrared and Raman Spectroscopy of Biological Materials*; CRC Press: Boca Raton, FL, USA, 2021.
4. Itkonen, J.; Annala, A.; Tavakoli, S.; Arango-Gonzalez, B.; Ueffing, M.; Toropainen, E.; Ruponen, M.; Casteleijn, M.G.; Urtti, A. Characterization, Stability, and In Vivo Efficacy Studies of Recombinant Human CNTF and Its Permeation into the Neural Retina in Ex Vivo Organotypic Retinal Explant Culture Models. *Pharmaceutics* **2020**, *12*, 611, doi:10.3390/pharmaceutics12070611.
5. Cattell, R.B. The Scree Test For The Number Of Factors. *Multivariate Behavioral Research* **1966**, *1*, 245-276, doi:10.1207/s15327906mbr0102_10.
